# Bridged Conductive Nanofibrous Membrane Overcoming the Porosity‐Conductivity Trade‐Off for Electrothermal Air Purification

**DOI:** 10.1002/advs.202508650

**Published:** 2025-11-06

**Authors:** Xiaoxue Yao, Zhenwen Zhang, Wei Deng, Chuhan Feng, Qili Xu, Wenzhu Lin, Zehua Peng, Yang Cao, Wang Guo, Bee Luan Khoo, Steven Wang

**Affiliations:** ^1^ Department of Mechanical Engineering City University of Hong Kong 83 Tat Chee Avenue, Kowloon Hong Kong 999077 China; ^2^ Department of Biomedical Engineering City University of Hong Kong 83 Tat Chee Avenue, Kowloon Hong Kong 999077 China; ^3^ School of Materials and Energy Guangdong University of Technology Guangzhou 510006 China; ^4^ Department of Mechanical Engineering The Hong Kong Polytechnic University Hung Hom, Kowloon Hong Kong 999077 China; ^5^ Department of Mechanical Engineering The University of Hong Kong Pokfulam Road Hong Kong 999077 China

**Keywords:** electrothermal, filtration, polypyrrole, porous conductive materials, sterilization

## Abstract

Emerging conductive porous materials hold remarkable promise for Joule‐heating applications like electothermal filtration, smart textiles, and energy management due to their porous and conductive synergy. However, their development is constrained by a design trade‐off between achieving high porosity for efficient flow transmission and maintaining a conductive network for effective charge transport. To overcome this, a bridged conductive nanofibrous membrane (BCNM) by linking polypyrrole‐coated nanofibers via self‐assembled polypyrrole nanowires is developed. This dual‐state network establishes continuous electron pathways while preserving multiscale porous channels, orchestrating air permeation, particulate capture, and electrothermal sterilization for all‐in‐one air purification. Leveraging this synergy, BCNM captures 98.79% of >0.3 µm particles under an ultra‐low pressure drop of 76 Pa and instantaneously self‐heats to 100 °C at low power to sterilize 99.49% of airborne bacteria. These performances compare favorably with leading conductive porous materials in both filtration performance and energy economy. A proof‐of‐concept solar‐powered purifier incorporating the BCNM outperforms existing purification technologies in terms of filtration, sterilization, energy efficiency, and cost. This work offers an innovative material–structure–function paradigm for developing energy‐interactive porous materials, with broad potential in smart filtration, biomedical protection, and sustainable energy systems.

## Graphical abstract

To overcome the porosity‐conductivity trade‐off in conductive porous membranes, a conductive nanofibrous membrane bridged by self‐assembled PPy nanowires is constructed, integrating multiscale porosity with continuous electron pathways. This material‐structure‐function strategy achieves low‐resistance and high‐efficiency filtration combined with electrothermal sterilization, offering an energy‐efficient, all‐in‐one solution for advanced air purification with sustainable pathogen control.

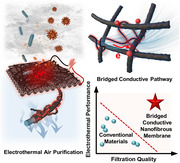

## 1 Introduction

Porous materials have long played a central role in energy and environmental fields such as filtration, separation, catalysis, and mass transfer, owing to their high surface area, tunable transport properties, and excellent interfacial control.^[^ ^1^, ^2^ ^]^ As materials evolve toward smart and multifunctional, a new class of conductive porous materials has emerged by incorporating electrical conductivity into these permeable structures. This integration allows them to support efficient mass transport of guests while offering dynamic functionalities such as electrothermal conversion, electrochemical activity, and stimuli‐responsive behavior.^[^ ^3^, ^4^, ^5^ ^]^ Among these, Joule heating is a built‐in feature of the conductive matrix to implement active thermal functionalities when an electric current passes.^[^ ^6^ ^]^ From an energy perspective, such localized surface heating enhances heat exchange when fluids pass through the conductive porous material, delivering superior thermal coupling compared to bulk heating.^[^ ^7^ ^]^ These combined characteristics make conductive porous materials attractive for electrothermal air purification, wearable thermal management, all‐weather desalination, and sustainable energy utilization.^[^ ^8^, ^9^, ^10^, ^11^ ^]^ This transition from static to active platforms is redefining how porous materials are designed for next‐generation applications.

Despite their promising potential, porous conductive materials face a fundamental design dilemma, where voids tend to reduce the effective charge carrier density and create more interface scattering to slow carrier mobility.^[^ ^12^ ^]^ Especially when the porous framework is bottom‐up composed of fibers, particles, or nanosheets, high porosity reduces the number of contact points between these units, thereby increasing contact resistance.^[^ ^13^ ^]^ Conversely, denser conductive units can improve conductivity but deteriorate mass transport efficiency.^[^ ^14^ ^]^ This competition is particularly pronounced in electrothermal air filtration. For instance, graphene‐stacked filters show outstanding heating efficiency and high filtration efficiency, yet their sub‐10 nm pore sizes pose an ultra‐high pressure drop of 800 Pa.^[^ ^15^ ^]^ To alleviate wind resistance, copper nanowires are loosely interwoven into an electrothermal mask via vacuum filtration, which lowers the pressure drop to 85 Pa, but is challenged by reduced heating efficiency and poor filtration.^[^ ^16^ ^]^ Electrospinning is a scalable and versatile approach for fabricating porous membranes with high surface area and tunable morphology.^[^ ^17^ ^]^ To impart electrical conductivity, conductive fillers like carbon nanotubes or graphene are usually blended into electrospun precursors, but their high concentrations often disrupt rheology and charge balance of jets, impairing fiber formation.^[^ ^18^ ^]^ As an alternative, post‐processing methods, including dip‐coating, deposition, and in situ polymerization, are employed to form conformal conductive shells on nanofibers. Nonetheless, these conductive fibers still lack interconnections, and the overall electrical conductivity remains poor.^[^ ^19^ ^]^ These limitations highlight the need for an advanced structural design strategy to construct a highly conductive scaffold without compromising the airflow permeability of porous conductive materials.

**Figure 1:**
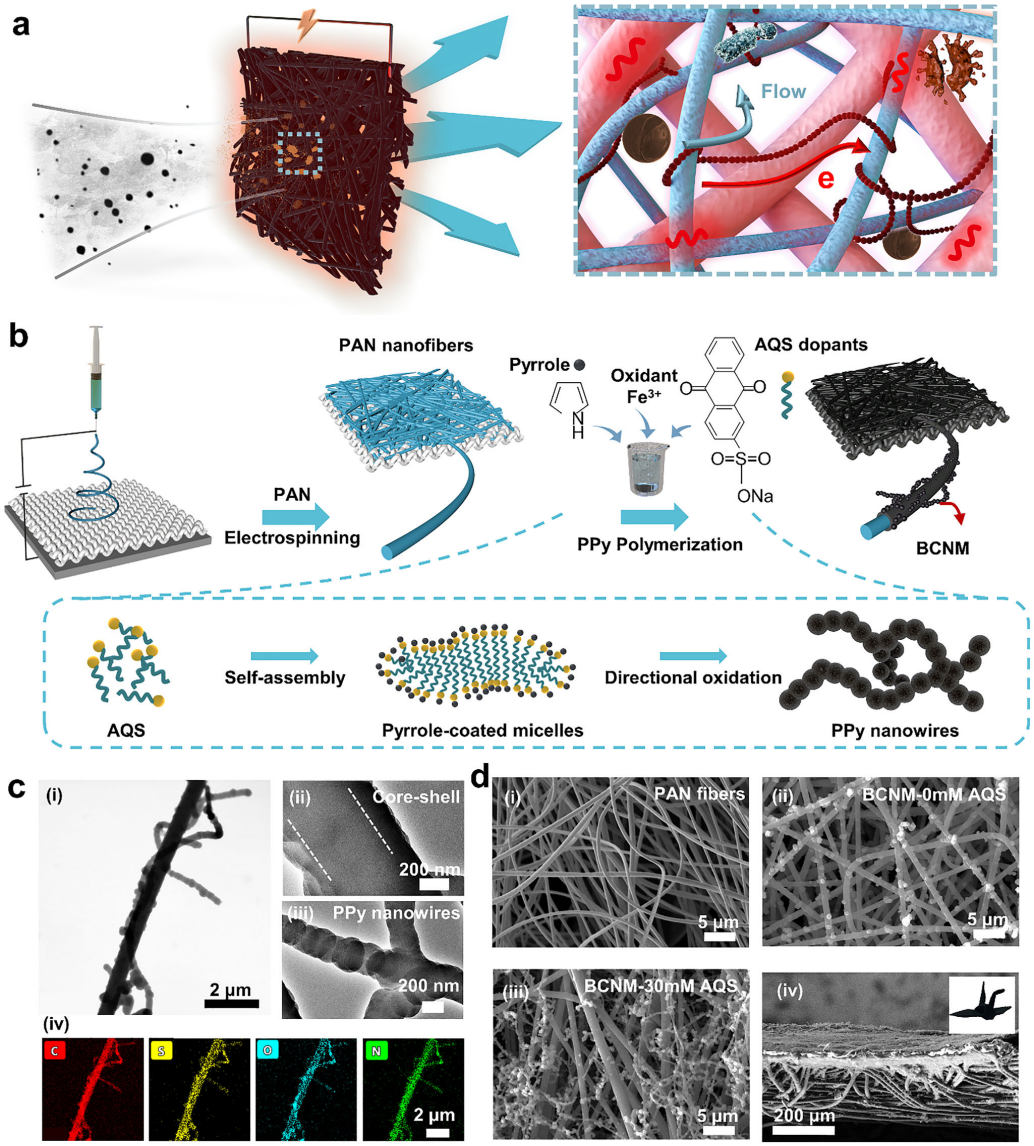
Design and characterization of BCNM. a) Schematic illustration of BCNM for electrothermal germicidal filtration. b) Fabrication process of BCNM. c) TEM images of i) a single fiber of BCNM composed of ii) PAN@PPy core‐shell structure and iii) PPy nanowires. iv) Element mapping images of the fiber. d) Top‐surface SEM images of i) pure PAN nanofibrous membrane, ii) BCNM fabricated without surfactant, and iii) BCNM fabricated with 30 mm surfactant. iv) Cross‐section SEM image of BCNM with flexibility demonstration in the inset.

Here, we propose a bridged conductive nanofibrous membrane (BCNM) that effectively addresses the long‐standing trade‐off between porosity and conductivity in conductive porous materials by constructing a dual‐state polypyrrole (PPy) network. This strategy leverages dopant concentration control to simultaneously achieve uniform deposition of PPy coatings on electrospun nanofibers and the self‐assembly of PPy nanowires, which bridge adjacent fibers and form a continuous multiscale conductive framework. This architecture preserves hierarchical porous pathways for simultaneous air filtration and electrothermal sterilization (**Figure** **1**a). Comprehensive evaluations are performed to compare the filtration efficiency, electrothermal conversion efficiency, and antibacterial performance of BCNM with existing conductive porous filtration materials. Furthermore, a solar‐powered BCNM air purifier prototype confirms its application potential for efficient, low‐energy indoor air purification. This innovation redefines conductive porous material design by overcoming the permeability–conductivity trade‐off, paving the way for advanced performance in environmental, biomedical, and clean energy applications.

## 2 Results and Discussion

### 2.1 Fabrication and Characterization

BCNM was fabricated by first electrospinning polyacrylonitrile (PAN) nanofibers onto a polyethylene terephthalate (PET) nonwoven substrate, followed by anthraquinone‐2‐sulfonic acid sodium salt (AQS)‐assisted polypyrrole (PPy) polymerization on both PAN nanofibers and PET microfibers (Figure 1b). Fourier‐transform infrared (FTIR) spectra verified successful PPy polymerization through characteristic peaks at 1542, 1328, and 1026 cm^−1^ corresponding to C═C stretching, pyrrole ring breathing vibration, and N–H in‐plane deformation, respectively. The emergence of a peak at 1184 cm^−1^ attributed to S = O stretching validated the incorporation of sulfonic surfactants.^[^ ^20^ ^]^ (Figure S1, Supporting Information) Transmission electron microscopy (TEM) and elemental mapping images revealed a conformal PPy shell uniformly coated on the PAN nanofiber, accompanied by pearl necklace‐like PPy nanowires entwining the fibers (Figure 1c). The dual‐scale conductive network originates from a micelle‐templated polymerization mechanism, where pyrrole monomers adsorb onto PAN fibers and undergo oxidative polymerization to form a uniform PPy coating. Meanwhile, excess AQS dopants self‐assemble into rod‐like micelles that confine pyrrole chain growth, directing the formation of 1D PPy nanowires (Figure 1d).^[^ ^21^ ^]^ As experimentally observed, with increasing AQS concentration from 0 to 30 mm, PPy evolved from nanoparticles to nanowires, boosting conductivity from 103.58 to 194.93 S m^−1^ by providing additional electron transport pathways (Figure S2, Supporting Information). Benefiting from conjugated chain extension and 1D ordering,^[^ ^22^, ^23^, ^24^ ^]^ the AQS‐induced nanowire network achieves even higher conductivity than the dense sodium dodecyl sulfate‐templated PPy coating, while at the same time preserving inter‐fiber porosity for superior air permeability (Figure S3, Supporting Information).

The integration of PET@PPy microfibers (≈12.7 µm), PAN@PPy nanofibers (≈500 nm), and PPy nanowires (≈244 nm) further established a hierarchical porous architecture, as evidenced by the mean pore size shrinking from 8.66 µm of the PET substrate to ≈0.58 µm after PAN nanofiber deposition and further to ≈0.40 µm with PPy nanowire bridging in the Scanning electron microscopy (SEM) images (Figure 1d‐iv; Figure S4, Supporting Information). Such pore distribution was also verified by the mercury intrusion method. As shown in Figure S5a (Supporting Information), both the PAN membrane and BCNM contain pores below 1 µm. For BCNM, the pore‐size distribution shifts slightly toward smaller sizes, and a peak appears ≈0.34 µm, consistent with the SEM statistics. Meanwhile, the porosity increases from 77.97% of the PAN membrane to 84.27% of the BCNM, indicating that PPy bridging improves channel connectivity. Brunauer–Emmette–Teller analysis also indicated a reduction in average pore diameter compared with PAN membranes, reflecting the smoother nanoscale surface created by uniform PPy coverage (Figure S5b, Supporting Information). This hierarchical pore framework creates interconnected capillary networks that impart rapid superhydrophilicity, enabling complete absorption of 10 µL water within 4 s to promote aerosol capture (Figure S6a and Video S1, Supporting Information).^[^ ^25^ ^]^ Meanwhile, the intertwined nanowires and nanofibers distribute stress efficiently, endowing BCNM with both strength (11.18 MPa) and flexibility (40% elongation), allowing it to be folded into complex shapes such as a paper crane (Figure S6b, Supporting Information). In addition, thermogravimetric analysis confirmed thermal stability up to 350 °C of BCNM, underscoring its suitability for high‐temperature air purification and electrothermal applications (Figure S6c, Supporting Information).

### 2.2 Air Filtration Performance

**Figure 2:**
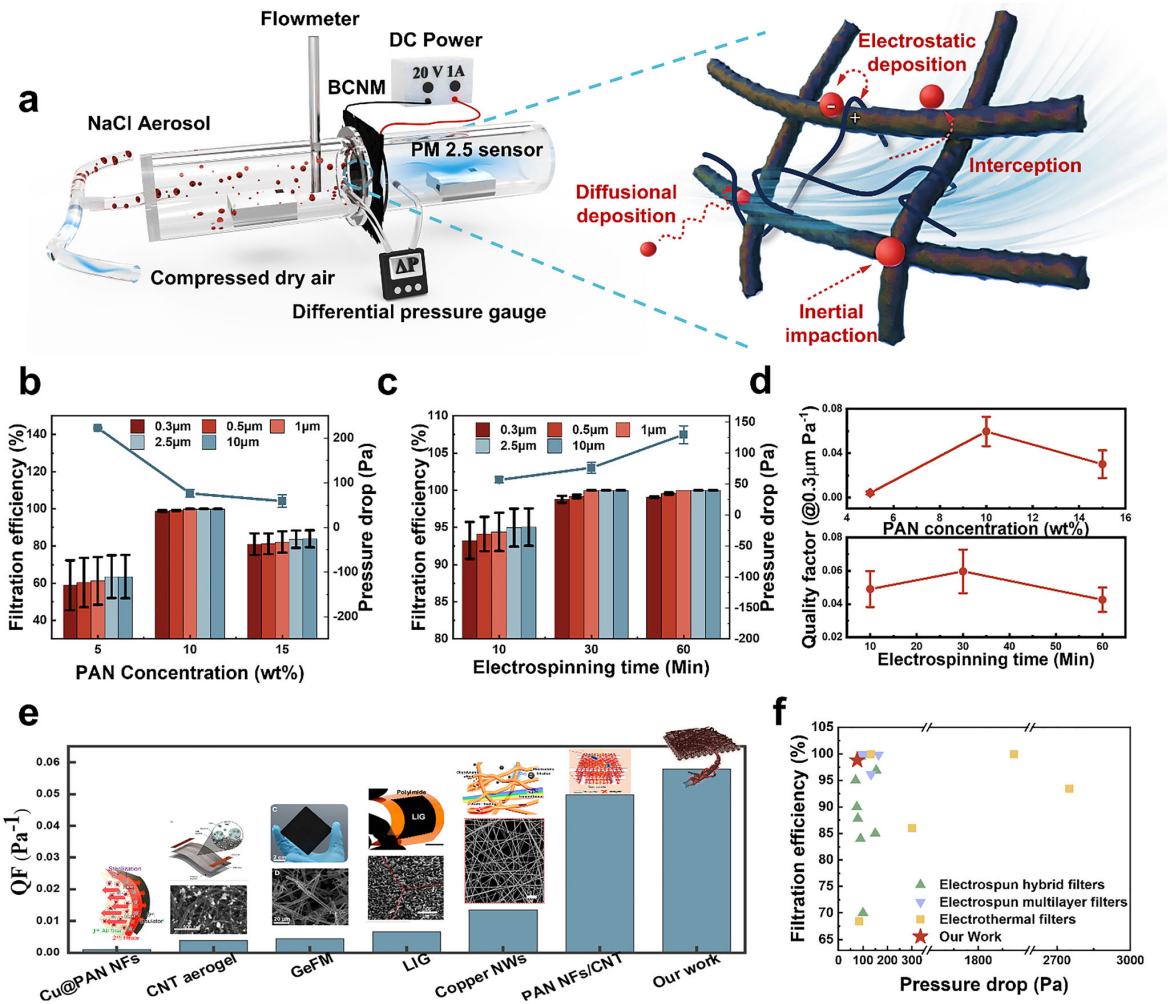
Air filtration performance of BCNM. a) Schematic illustration of the air filtration test and the filtration mechanism of BCNM. b) Impact of electrospinning concentrations and c) electrospinning times for BCNM filtration efficiencies and pressure drops under 30 L Min^−1^. Data are presented as the mean values ± standard deviations (*n* = 3 independent samples) d) QF of BCNMs for >0.3 µm particles. e) QF comparison of BCNM with present electrothermal air filters. f) Filtration efficiency and pressure drop comparison of BCNM with previously reported nanofibrous air filters. Due to variations in testing methods and parameters, the source data are provided in Table S1 (Supporting Information) for clarity.

To optimize the filtration performance of BCNM, membranes were fabricated using various electrospinning parameters and systematically tested in a homemade air filtration testing apparatus by injecting NaCl aerosols mixed with clean and dry compressed air at an airflow rate of 30 L Min^−1^. A differential pressure gauge and two calibrated particle counter sensors (Figure S7, Supporting Information) were employed to monitor real‐time pressure drop and particle concentration across the membrane under a controlled environment (**Figure** **2**a). The filtration efficiency *η* is determined using the formula:^[^ ^26^ ^]^

(1) $$\eta =\left(1-\frac{C_{out}}{C_{in}}\right)\;\times 100\%$$

where *C_in_* and *C_out_* represent the particle concentrations at the inlet and outlet tubes, respectively. The measurement results shown in Figure 2b,c, as the PAN concentration increased from 5, 10 to 15 wt.%, the average fiber diameter enlarged from 261, 469 to 709 nm, accompanied by higher porosity and lower packing density (Figure S8, Supporting Information), alleviating the pressure drop from 223, 76 to 59 Pa. In contrast, the capture efficiency of >0.3 µm particles varied in a nonmonotonic trend, rising from 58.88% to 98.79%, then dropping to 81.05%. This behavior is attributed to fiber morphologies, where low concentrations produce bead defects that disrupt fiber continuity, whereas high concentrations introduce oversized pores, both causing fine particles to escape. At a fixed PAN concentration of 10 wt.%, extending spinning time from 10, 30 to 60 min increases the packing density without fiber diameter and pore size change (Figure S9, Supporting Information), leading to a filtration improvement from 93.26%, 98.79% to 99.06% but a corresponding rise in pressure drop from 57, 76, to 130 Pa.

To comprehensively evaluate filtration performance, the filtration quality factor (QF) was calculated to assess the trade‐off between filtration efficiency and pressure drop by the following formula:^[^ ^26^ ^]^

(2) $$QF=\frac{-\ln\left(1-\eta \right)}{\Delta P}$$

where *η* represents the particulate filtration efficiency and *∆P* denotes the pressure drop across the porous samples. As shown in Figure 2d, the BCNM electrospun from 10 wt.% PAN for 30 min with 98.79% filtration efficiency and 76 Pa pressure drop achieved the highest QF up to ≈0.06 Pa^−1^, which compares favorably with reported electrothermal air filters (Figure 2e). Due to differences in aerosol types and testing protocols, detailed comparisons are referenced in Table S1 (Supporting Information). Because humid or charged aerosols may lead to an overestimation of efficiency, the best‐performing BCNM was further examined using a TSI 8130A tester with a dryer and neutralizer under the standard face velocity (32 L min^−1^, 5.33 cm s^−1^).^[^ ^27^ ^]^ After aerosol neutralization, BCNM still achieved 98.51% PM0.3 efficiency, confirming robust intrinsic filtration performance, which can be understood from its particle capture mechanisms. Like other nanofibrous membranes, the particle capture mechanisms of BCNM involve inertial impaction, interception, diffusion, and electrostatic attraction.^[^ ^26^ ^]^ However, the conductive PPy coating dissipates charges, which weakens electrostatic adsorption.^[^ ^28^ ^]^ Consequently, the filtration efficiency of BCNM decreased slightly from 98.76% to 97.74% when its surface potential was eliminated from +0.08 kV to zero (Figure S10, Supporting Information). The weak electrostatic effect likely originates from the positively charged PPy backbone, which was later confirmed by zeta potential measurements.^[^ ^29^ ^]^ Predominantly governed by mechanical filtration, BCNM nevertheless achieves both high efficiency and low resistance, which can be attributed to gradient pore structure and high porosity (Figure 2f). In this architecture, the PET microfiber layer with large micropores functions as a prefilter inhibiting penetration of particles larger than 1 µm through inertial impaction, while the bridged nanoporous network captures submicron particles mainly via interception and diffusion. Meanwhile, the bridging strategy generates fine pores without significantly increasing membrane thickness but enhancing porosity, thereby ensuring excellent breathability.^[^ ^30^ ^]^ The hierarchical effectiveness is also supported by COMSOL simulations showing a fine particle filtration rate of 90.34%, significantly outperforming single‐scale membranes of 45.75% (Figure S11 and Note S3 I, Supporting Information).

### 2.3 Electrothermal Performance

**Figure 3:**
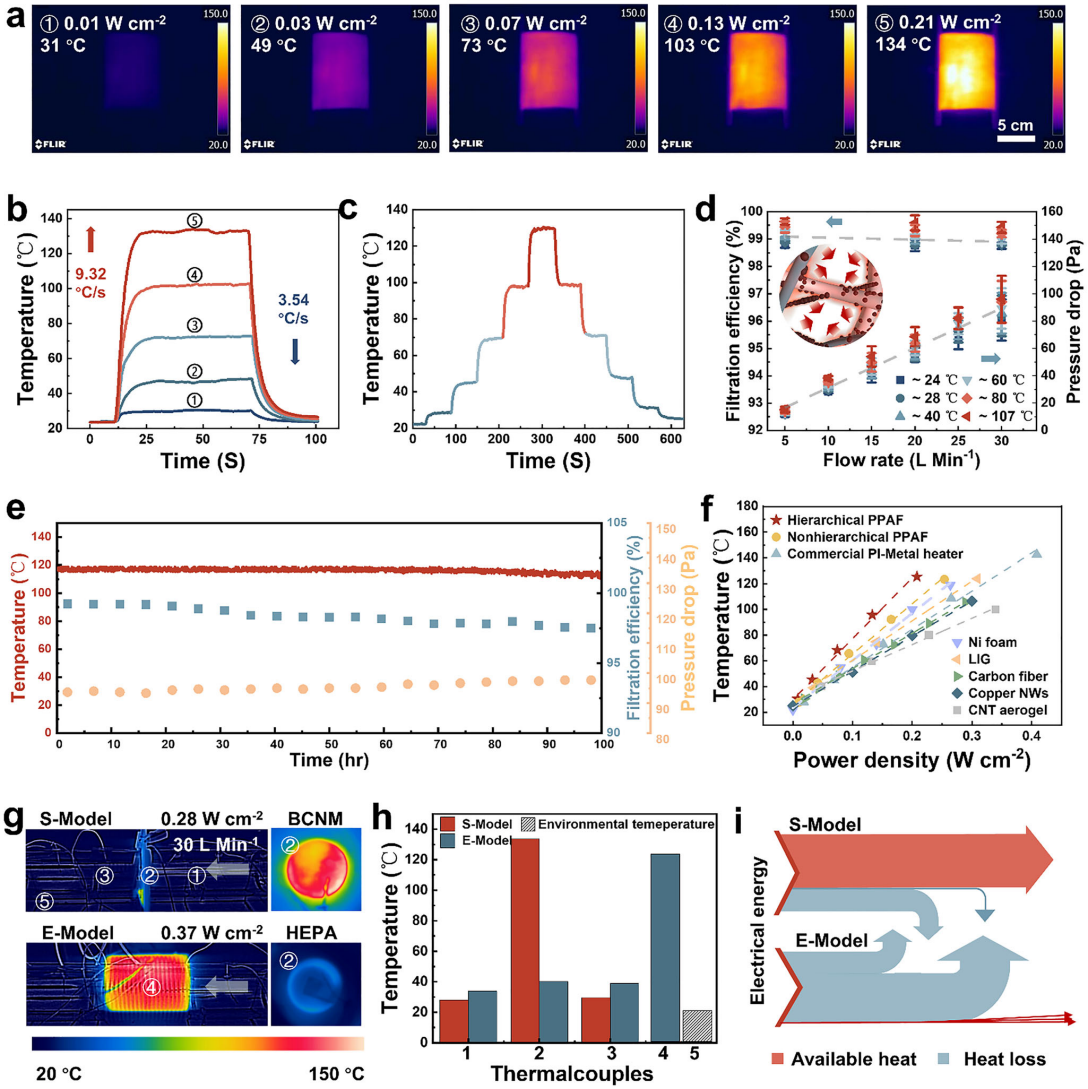
Electrothermal performance of BCNM. a) Temperature profile variance and b) temperature change curve of BCNM applied with gradient powers. c) Stepwise voltage‐responsibility of BCNM. d) Pressure drops and filtration efficiencies across BCNM under different flow rates and electrothermal temperatures. Data are presented as the mean values ± standard deviations (*n* = 3 independent samples). The inset is a schematic illustration of the pore shrinkage phenomenon when heating. e) Electrothermal performance (red line), filtration efficiency (blue square dot), and pressure drop (yellow circle dot) of Joule‐heated BCNM (powered by 0.17 W cm^−2^) during 100 h aging under high‐humidity conditions (25 °C, 90%RH). f) Comparison of the electrothermal conversion efficiency between hierarchical BCNM, homogeneous BCNM, a commercial heater, and other conductive porous materials. g) Infrared thermal images of a self‐heating BCNM (S‐Model) and an external‐heating HEPA filter (E‐Model) in an acrylic tube with airflow of 30 L Min^−1^. h) Temperature comparison of two models. i) Sankey diagram comparing the energy distribution of two models.

Beyond passive particle capture, the continuous conductive network of BCNM enables Joule heating. As shown in **Figure** **3**a, under stepwise increases in power density (0.01, 0.03, 0.07, and 0.13 W cm^−2^), the membrane rapidly reached steady‐state temperatures of 31, 49, 73, and 103 °C, with a front–back surface difference of less than 6 °C, confirming uniform heating across the structure. This is attributed to the inherent electrothermal capability of the PAN@PPy layer and the PET@PPy substrate, as well as the thermal conduction between them (Figure S12, Supporting Information). When the input was further raised to 0.21 W cm^−2^, BCNM exhibited exceptionally fast dynamics, heating to 134 °C at 9.32 °C s^−1^ and cooling to room temperature at 3.54 °C s^−1^, approximately seven and four times faster than a commercial PI‐Metal heater (Figure 3b; Figure S13a, Supporting Information). In addition, a linear current–voltage response indicated stable resistance during heating, effectively avoiding inrush currents and ensuring operational safety (Figure S13b, Supporting Information). Taken together, these features confer sensitive and controllable thermal responsiveness, as demonstrated by proportional temperature steps under stepwise power inputs (Figure 3c; Video S2, Supporting Information). This electrothermal responsiveness further translates into effective filtration performance. To verify this effect, we studied how electrothermal response influences filtration performance under different flow conditions (Figure 3d). With increasing airflow, the pressure drop rose proportionally while the filtration efficiency declined slightly due to reduced particle residence time and weaker diffusion.^[^ ^31^ ^]^ In contrast, elevating the membrane temperature enhanced both airflow resistance and particle removal owing to thermally induced pore constriction. For example, when heated to 107 °C, the removal efficiency of >0.3 and >2.5 µm particles increased to 99.43% and 99.997%, respectively, although the pressure drop also rose moderately to 96 Pa. Notably, in practical applications, heating the membrane to 60 °C is already sufficient for pathogen inactivation.^[^ ^32^, ^33^ ^]^ At this temperature, BCNM still maintained a high filtration efficiency of 98.95% with a pressure drop of only 86 Pa, demonstrating a favorable balance between performance and breathability.

Long‐term durability was also confirmed. In a 100‐h aging test under continuous Joule heating under 90% RH, the electrothermal temperature declined only from 117.3 to 112.9 °C, the filtration efficiency for >0.3 µm particles remained as high as 97.49%, and the pressure drop rose by merely 4 Pa (Figure 3e). Such stable performance can be attributed to the hierarchical porous structure of BCNM, which enables multistage particle retention while preserving overall porosity and ensuring sustained filtration efficiency. The minor reduction was mainly associated with weakened electrostatic attraction at high humidity and partial particle accumulation on fiber surfaces. As the electrothermal temperature declined, the electrical conductivity moderately decreased to 192.42 S m^−1^, corresponding to a drop of about only 3% (Figure S14a, Supporting Information). This minor change is attributed to an oxidation of PPy, as X‐ray photoelectron spectroscopy analysis showed a slight increase in oxygen content from 38.37% to 42.02%, whereas FTIR spectra displayed no discernible change in the C═C/C─C stretching vibration of PPy chains at 1531–1561 cm^−1^, indicating that the conjugated backbone of PPy remained intact (Figure S14b,c, Supporting Information).^[^ ^34^ ^]^ This stability is attributed to the synergistic effect of AQS doping and low‐temperature polymerization, which promotes ordered chain growth, enhances planarity, and hinders oxygen penetration.^[^ ^23^, ^35^, ^36^ ^]^ Strain‐stress mechanical testing also exhibited no detectable change after this 100‐h static aging test (Figure S14d, Supporting Information). Furthermore, dynamic stability was validated by cycling tests. After 2000 switching cycles, its electrothermal temperature decreased by only 4.4% (Figure S15, Supporting Information). Moreover, after 2000 bending cycles, BCNM still maintained ≈104 °C in the flat state and ≈87 °C in the bent state, with no signs of delamination, cracks, or interlayer separation observed by SEM (Figure S16, Supporting Information). Collectively, these results demonstrate that BCNM maintains excellent long‐term electrothermal performance, filtration efficiency, and structural robustness under both static and dynamic conditions.

Following Joule's law, the electrothermal conversion efficiency was quantified by fitting the relationship between temperature and power density (Note S1, Supporting Information). As shown in Figure 3f, the hierarchical BCNM had the steepest slope of 473.54 °C cm^2^ W^−1^, which is higher than that of non‐hierarchical BCNM, PI–metal heaters, and other porous heaters reported previously.^[^ ^15^, ^16^, ^37^, ^38^, ^39^ ^]^ This outstanding efficiency originates from the intrinsic polymeric nature and the bridged porous structure of BCNM, which together provide a low thermal conductivity (≈0.08 W m^−1^ K^−1^) to suppress heat dissipation, combined with high electrical conductivity that ensures homogeneous Joule heating.^[^ ^40^, ^41^ ^]^ Simulations further validated that, compared with high‐thermal‐conductivity CNT networks (≈2000 W m^−1^ K^−1^), BCNM achieved a maximum temperature 34.2% higher, confirming its superior heat‐concentration capability (Figure S17 and Note S2, Supporting Information). To validate this advantage at the dynamic system level, we compared a self‐heating BCNM filter (S‐Model) with a high efficiency particulate air (HEPA) membrane heated externally (E‐Model) under different ventilation conditions (airflow rates from 30 to 70 L Min^−1^, 25 °C‐60% RH and 30 °C‐85% RH). Under 30 L min^−1^ airflow at 20 °C and 60% RH, BCNM rapidly reached 135 °C with only 0.28 W cm^−2^ input, whereas the HEPA membrane surface warmed to merely 40 °C when its external heater was heated to 123 °C by a higher power input of 0.37 W cm^−2^ (Figure 3g,h). Across all airflow rates, the S‐Model maintained a downstream ΔT advantage of 8.9–10.3 °C over the E‐Model. Even under hot and humid conditions, it maintained a downstream temperature of 36.59 °C compared to 48.02 °C for the E‐Model, highlighting the robustness of localized Joule heating for energy focusing and thermal comfort (Figure S18, Supporting Information). COMSOL simulations supported this observation, showing localized heating above 140 °C in BCNM compared with diffuse heating below 45 °C in HEPA (Figure S19 and Note S2, Supporting Information). Heat transfer analysis further revealed that 62.3% of electrothermal energy in BCNM was effectively transferred via airflow convection, while only 0.78% reached the HEPA membrane in the E‐Model (Figure 3i; Figure S20, Supporting Information). These results demonstrate that BCNM combines high filtration efficiency with system‐level energy focusing.

### 2.4 Active Germicidal Air Purification Performance

**Figure 4:**
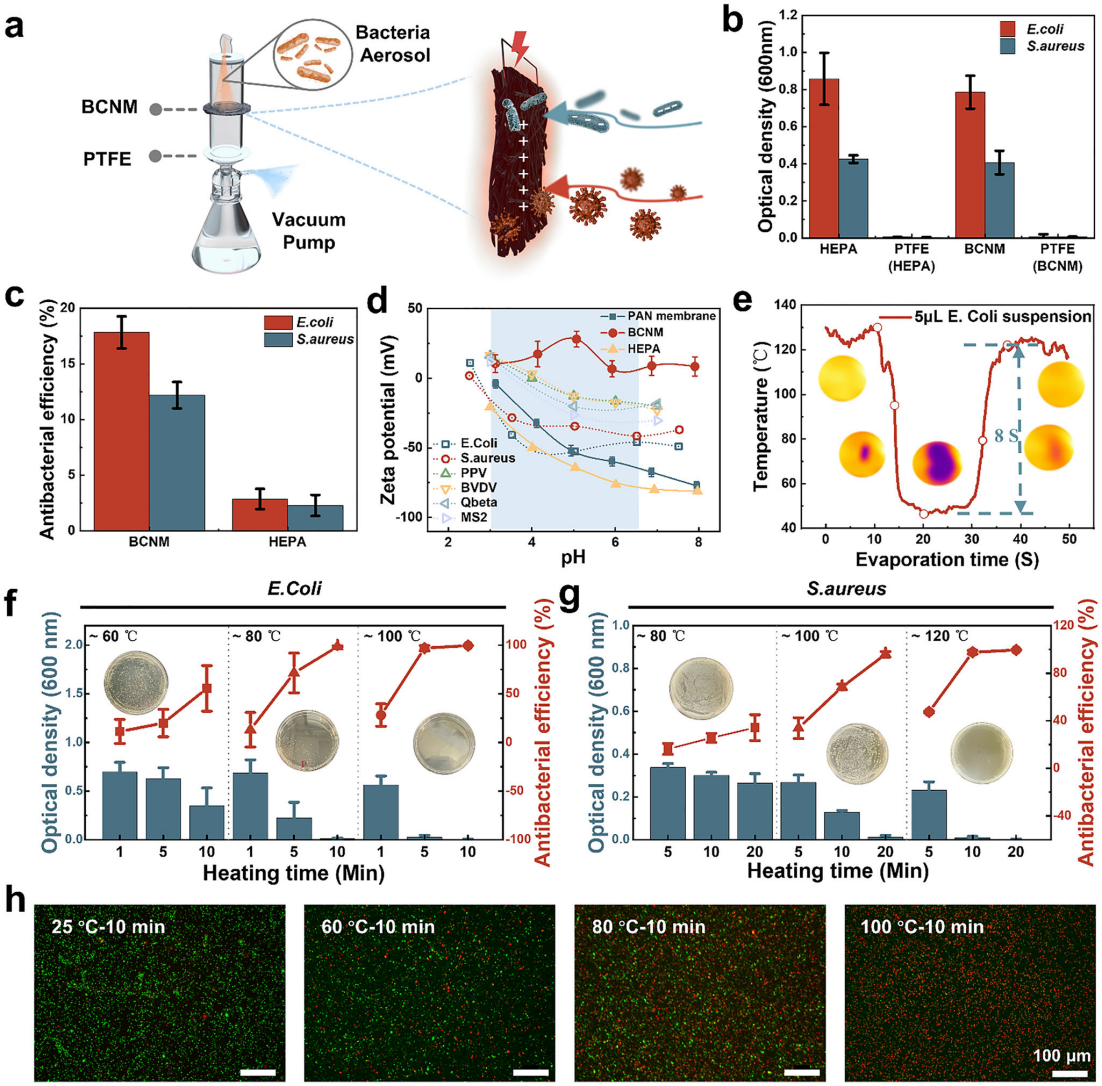
Electrothermal air sterilization performance of BCNM. a) A schematic illustrates the test setup and sterilization method of BCNM. b) OD600 comparison of bacteria grown on HEPA filters and BCNM, as well as their respective PTFE layers. c) Nonthermal antibacterial efficiency of BCNM and HEPA filtration membrane. d) Zeta potential comparison of pure PAN membrane, BCNM, and HEPA filters with typical pathogens under various pH conditions. e) Evaporation temperature curve with time of a 5 µL *E. Coli* suspension droplet on electrothermal BCNM. f) Optical density change of *E. Coli* and g) *S. aureus* grown on BCNMs heated by gradient temperatures for different durations (Blue bars); Red dots represent corresponding thermal antibacterial efficiencies; Insets are the distribution of bacterial colonies on agar plates heated at gradient temperatures for 10 min. h) Epifluorescence images of live/dead dyed *E. Coli* on BCNM when electrothermally treated at a series of temperatures after 10 min. Error bars represent the standard deviation from three independent samples.

The electrothermal filtration capability of BCNM provides a foundation for thermo‐sterilizing air purification. To evaluate antibacterial performance, aerosolized infectivity trials were conducted using gram‐negative Escherichia coli (*E. coli*) and gram‐positive Staphylococcus aureus (*S. aureus*) with a multi‐stage setup as illustrated in **Figure** **4**a. In these trials, aerosolized bacteria were captured by either Joule‐heated BCNM or commercial H13 HEPA membranes, while penetrated bacteria were collected on downstream PTFE filters. After recovery and culture, OD600 measurements confirmed comparable filtration performance (Figure 4b). By calculation, BCNM achieved efficiencies of 98.93% for *E. coli* and 98.62% for *S. aureus*, which were close to the 99.27% and 99.06% obtained with HEPA membranes. Beyond passive capture, BCNM exhibited intrinsic antibacterial activity. Static contact tests demonstrated antibacterial rates of 17.82% for *E. coli* and 12.18% for *S. aureus*, which were far higher than those of HEPA membranes that remained below 3% (Figure 4c). Agar plate culture results supported the antibacterial activity of BCNM in the absence of heat (Figure S21, Supporting Information). This effect can be attributed to the PPy coating. As a cationic conductive polymer, PPy carries delocalized positive charges that interact electrostatically with negatively charged bacterial membranes, partially disrupting their integrity.^[^ ^29^ ^]^ Zeta potential measurements revealed that BCNM exhibited a positive surface potential, whereas HEPA membranes showed negative values (Figure 4d). Combined with the fact that most pathogens possess negatively charged surfaces, this finding further supports the proposed mechanism.^[^ ^42^, ^43^, ^44^, ^45^ ^]^

When activated electrothermally, BCNM demonstrated strong germicidal efficacy. Heating to 60 °C for 5 min already inactivates ≈20% of *E. coli*. Raising the temperature to 100 °C increased the inactivation to more than 96.85%, and extending the treatment to 10 min at the same temperature raised it further to 99.48%, as reported in Figure 4f. In comparison, *S. aureus* displayed higher thermal resistance. At 80 °C, only 34.25% of the bacteria were inactivated after 20 min, whereas at 100 and 120 °C the rates rose to 95.94% and 97.65%, respectively, eventually reaching 99.49% after 20 min at 120 °C, as illustrated in Figure 4g. This difference is consistent with their structural features as the thicker peptidoglycan wall of *S. aureus* provides greater protection, while the thinner, protein‐rich outer membrane of *E. coli* is more vulnerable to electrostatic disruption and thermal denaturation.^[^ ^46^ ^]^ Supporting evidence from agar plating and fluorescence staining confirmed progressive cell death with increasing electrothermal temperature, as shown in Figure 4h. It is worth noting that the high humidity (>99% RH) of the test aerosols likely prolonged sterilization times compared to typical indoor environments (<60% RH), where faster inactivation is expected.^[^ ^47^ ^]^ Supporting this, a 5 µL bacterial suspension was dropped on Joule‐heated BCNM at 120 °C, where it was fully absorbed due to its superhydrophilicity and evaporated within 8 s, demonstrating strong potential for rapid inactivation of real aerosols (Figure 4e).

**Figure 5:**
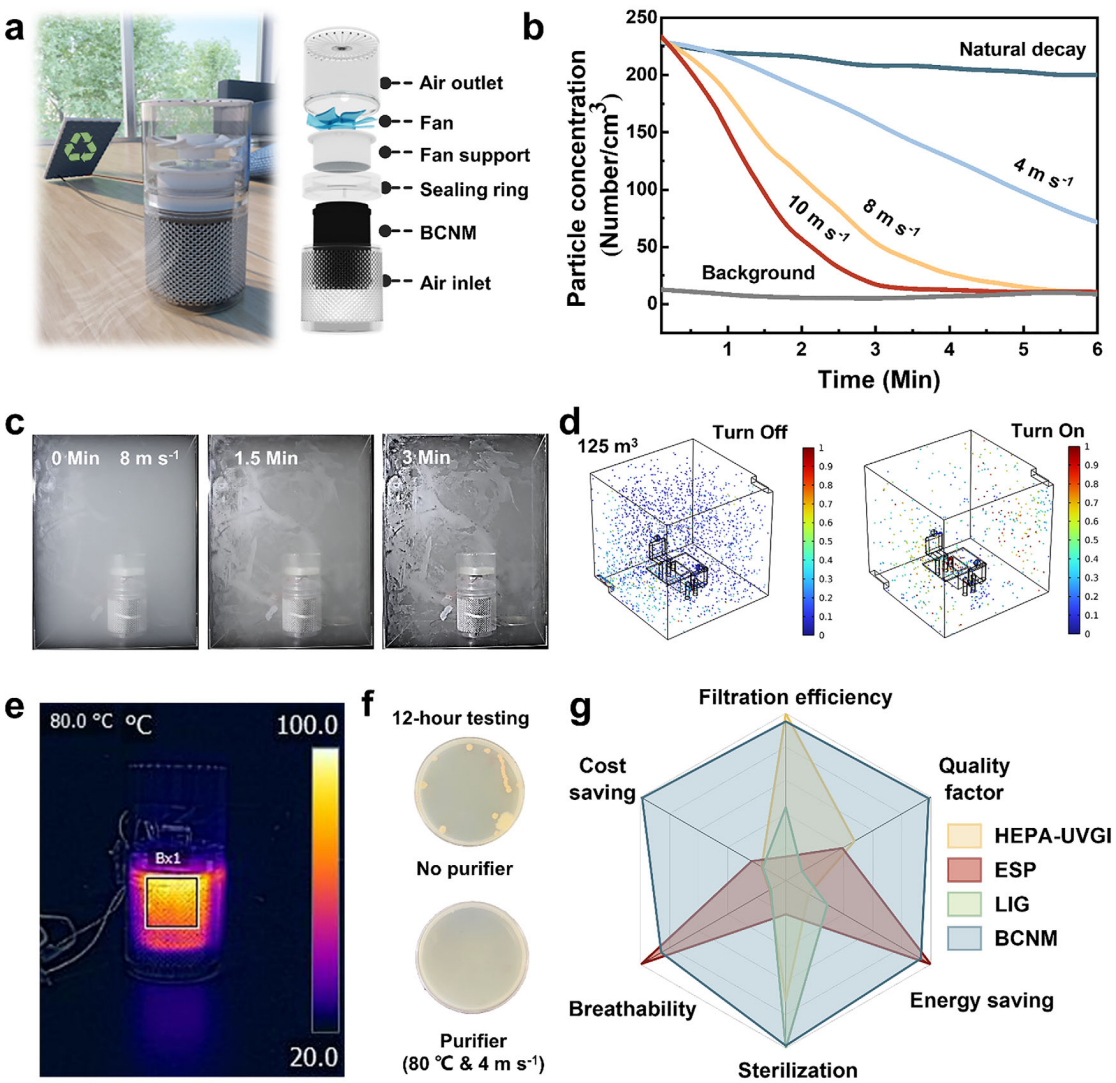
Air purification performance of a BCNM‐purifier. a) Schematic illustration of a solar‐powered BCNM‐purifier for indoor air purification with components illustration of the purifier. b) Filtration efficacy comparison of the purifier under different airflows adjusted by the fan in a 55 L box. c) Time‐lapse of pollution purification process after starting the purifier at wind velocity of 8 m s^−1^. d) Simulated comparison of particle distribution at 200 s in a larger room when turning on and off the BCNM‐purifier. e) infrared thermal picture of the working purifier. f) Colony distribution on agar plates with and without the purifier in a room after 12 h of testing. g) Radar plot compares the performance of BCNM with the commercial HEPA‐UVGI system, ESP system, and LIG electrothermal air filter; Source data are provided in Tables S2 and S3 (Supporting Information).

To bridge the gap between material‐level properties and real‐world functionality, a BCNM‐based air purification prototype was developed to evaluate its thermal air sterilization performance. The freestanding device integrates a cylindrical BCNM membrane, a brushless DC fan, and a 3D‐printed casing (**Figure** **5**a). For sustainable, all‐weather operation, the device was powered by an integrated off‐grid system including a solar panel, rechargeable battery, controller, and voltage regulator (Figure S22, Supporting Information). Filtration tests were conducted in a 55 L sealed chamber using incense smoke as a pollutant. As fan airflow increased from 0 to 10 m s^−1^, the particle removal velocity improved from 12.07% to 95.48% (Figure 5b). At 8 m s^−1^, most 0.3 µm particles were removed in an exponential decay within 3 min, which is also visualized in a time‐lapse smoke clearance experiment (Figure 5c; Video S3, Supporting Information). Complementary simulations in a larger 125 m^3^ room model confirmed that the purifier doubled aerosol removal efficiency at 1 m s^−1^ compared to passive conditions (Figure 5d, Figure S23, and Note S3 III, Supporting Information). To assess disinfection performance, this BCNM purifier was operated with Joule heating of 80 °C in a 30 m^2^ room for 12 h (Figure 5e). Agar plates exposed during the test showed no detectable bacterial colonies after incubation, whereas noticeable proliferation occurred in the control group when the purifier was turned off. This result confirms the strong potential of BCNM for air sterilization in real‐world environments (Figure 5f).

To better outline the characteristics and performance of BCNM for real applications, it is instructive to compare it with existing filtration–sterilization technologies. Electret filters such as MERV 15–16 or HEPA media combine low resistance with excellent capture efficiency, but lack intrinsic sterilization and are prone to charge decay or microbial proliferation in humid environments.^[^ ^48^, ^49^ ^]^ To compensate, these filters are often coupled with ultraviolet germicidal irradiation (HEPA‐UVGI), which enhances disinfection but introduces high energy consumption and potential UV‐related health risks.^[^ ^50^ ^]^ Electrostatic precipitators (ESPs) typically used in industries are known for their low airflow resistance and energy efficiency, yet they are struggling with low particle capture efficiency and harmful ozone generation.^[^ ^51^ ^]^ The first electrothermal air filter made from laser‐induced graphene (LIG) offers self‐heating sterilization capability, but its ultrafine pore sizes and densely packed structures often lead to limited breathability.^[^ ^15^ ^]^ In contrast, BCNM unifies mechanical filtration and electrothermal sterilization within a single lightweight porous membrane, enabling both efficient capture and active inactivation while preserving breathability and long‐term stability. This integrated material–structure–function design allows BCNM to outperform current technologies in efficiency, breathability, sterilization capability, energy economy, and cost‐effectiveness, establishing it as a structurally rational and practically scalable solution for next‐generation sustainable air purification (Figure 5g; Tables S2 and S3, Supporting Information).

## 3 Conclusion

In summary, we developed a bridged conductive nanofibrous membrane that overcomes the long‐standing trade‐off between air filtration and electrical properties in electrothermal porous materials. Through a dual‐state PPy network, BCNM delivers high >0.3 µm particles filtration efficiency (98.8%) under an ultra‐low pressure drop of 76 Pa, along with ultrafast Joule heating to 100 °C within 12 s and high electrothermal efficiency of 473.54 °C cm^2^ W^−1^. In a simulated ventilation duct, it generates up to 80 times available heat than externally heated HEPA filters and maintains excellent thermal and filtration stability over a 100 h aging test. The synergistic combination of aerosol filtration and localized heating enables 99.5% bacterial aerosol inactivation within 10 min. A solar‐powered BCNM purifier further demonstrated effective aerosol removal and room‐scale sterilization. Compared with conventional multi‐module sterilizing filters, BCNM offers advantages by integrating filtration and sterilization into a single and portable platform. This rational structure‐to‐function pathway offers not only a high‐performance solution for energy‐efficient air purification but also a versatile framework for designing next‐generation conductive porous materials applied in applications of biomedical engineering, environmental remediation, and energy generation.^[^ ^52^, ^53^, ^54^, ^55^ ^]^

## 4 Experimental Section

### Preparation of BCNM

PAN solutions with gradient concentrations (5%, 10%, 15%) were prepared by dissolving PAN powder (Mw = 150000) in DMF under stirring at 80 °C for 5 h. Electrospinning was performed using an HZ‐11 setup, ejecting the solution at 0.5 mL h^−1^ under 20 kV onto a PET substrate (filtration efficiency ≈4% @300 nm, pressure drop <15 Pa @30 L min^−1^). By adjusting deposition times (10–60 min), nanofibrous membranes with controlled basis weights were obtained. For PPy coating, PAN membranes were immersed in an ice‐cold solution containing 0.18 m pyrrole and 30 mm anthraquinone‐2‐sulfonic acid sodium salt (AQS), followed by the dropwise addition of 0.4 m Fe(NO_3_)_3_ as oxidants. After 4 h of polymerization, the PAN@PPy membranes were rinsed and vacuum‐dried at 80 °C. All experiments were conducted at 23 ± 1 °C and 50 ± 5% RH.

### Air Filtration Evaluation of BCNM

A homemade acrylic tube system was used for the filtration performance test. The platform consisted of two acrylic tubes (D = 7 cm, L = 20 cm), with the test filter mounted between them. NaCl aerosol (23 ± 1 °C, 30 ± 2% RH) was generated by mixing particles from an aerosol generator (8026, TSI, USA) with clean, dry compressed air (JIAPU, China), and upstream and downstream concentrations were monitored using laser sensors (PMS9103M, PLANTOWER, China). For particles >0.3, >0.5, and >1 µm, the output unit was the number of particles per 0.1 L of air, whereas for >2.5 and >10 µm it was expressed as mass concentration (µg m^−3^). To minimize measurement bias, the upstream mass concentration of >2.5 µm particles was fixed at 480 ± 20 µg m^−3^, and the two sensors were calibrated by linear regression analysis before testing to ensure consistency (Figure S7, Supporting Information). The pressure drop across the test filter was measured with a differential pressure gauge (Testo 510, Germany), and the airflow rate was verified using an airflow probe (Testo 400, Germany). In addition, the PM_0_._3_ filtration efficiency was evaluated with an automated filter tester (8130A, TSI, USA), which generates dried and charge‐neutralized monodisperse NaCl aerosols with a mass median diameter of 260 nm and a count median diameter of 75 nm. According to EN779:2012 standards, the test airflow rate was fixed at 32 L min^−1^ (5.33 cm s^−1^). The surface potential of BCNM was measured by a static charge tester (JH‐TEST) and was then neutralized using an ionizing static eliminator (YUCHENGTECH) for the electrostatic attraction study.

### Electrothermal Analysis of BCNM

The 10 × 10 cm^2^ BCNMs were fixed by graphite rod electrodes for the electrothermal test. Electric power provided to the samples was controlled by a power source (PFS‐606D, FMDQGS, China). The temperature profile of electrothermal membranes was recorded by an infrared camera (EX‐8, FLIR, USA). The aging test was conducted in a climate chamber (LC‐QHX, LICHEN). The heat distribution of self‐heated BCNM and HEPA filter externally heated by a PI‐Metal heater (100 mm × 200 mm, 24 V/80 W, Keyiman, China) in the homemade acrylic tube system was measured by 5 thermocouples (34465A, Keysight, USA) and an infrared camera. Thermocouple 2 was mounted on the filters. The distance from inlet thermocouple 1 and outlet thermocouple 3 to the middle filters was 5 cm. Thermocouples 4 and 5 monitored the temperature of the PI‐Metal heater and environment, respectively.

### Self‐Heating Sterilization Test of BCNM

*E. coli* and *S. aureus* suspensions (1 × 10^^9^ CFU mL^−1^) were prepared for antibacterial tests. For static contact antibacterial tests, five pieces of 1 × 1 cm^2^ BCNM and commercial HEPA filtration membranes (Polypropylene/Polyethylene Terephthalate, H13, 99.97% @0.3 µm) were immersed in bacterial suspensions and incubated at 37 °C for 24 h. Then, the optical density at 600 nm (OD_600_) of the supernatant was measured using a microplate reader (SpectraMax 5, Molecular Devices, USA). Additionally, 2 µL of the culture was diluted and inoculated onto agar plates, which were incubated at 37 °C for 24 h to assess bacterial colony growth.

For the filtration and thermal disinfection test, suspensions were diluted to 5 × 10^^8^ CFU mL^−1^, aerosolized using a nebulizer (Air Pro vill, Feellife, China), and passed through the BCNM at 15 LPM using a vacuum pump (SN‐HP‐02, Sunne, China) for 10 min. Membranes were then Joule‐heated to 60, 80, and 100 °C for 1, 5, or 10 min (*E. coli*) as well as to 80, 100, and 120 °C for 5, 10, or 20 min (*S. aureus*), respectively. Unheated membranes served as controls. After heating, membranes were cut into 1 × 1 cm^2^ pieces, immersed in nutrient broth, and vortexed for 5 min to release captured bacteria. The broth was incubated at 37 °C for 24 h for OD_600_ measurement. Meanwhile, 2 µL of the culture was plated onto agar plates and incubated under the same conditions to evaluate bacterial growth. Moreover, the collected *E. Coli* were stained using NucGreen/EthD‐III dyes (EX3000, Solarbio, China), and fluorescence images of stained live/dead bacteria from each sample were captured using a fluorescence microscope (BX53, Olympus).

Antibacterial efficiency (𝜂_a_) was calculated as:

(3) $$\eta_{a}=\frac{OD_{\mathrm{cont}\mathrm{rol}}-OD_{\mathrm{samp}\mathrm{le}}}{OD_{\mathrm{cont}\mathrm{rol}}}\;\times 100\%$$

where, for static contact tests, 𝑂𝐷*_control_* is the OD_600_ of the original bacterial suspension, and 𝑂𝐷*_sample_* is that after incubation with membranes. For thermal disinfection tests, 𝑂𝐷*_control_* and 𝑂𝐷*_sample_* refer to the OD_600_ of cultures treated with unheated and heated BCNMs, respectively.

### Performance Evaluation of BCNM Purifier

All the supporting parts and the outer covers of the air purifier were prepared through 3D printing (1700 Pro SLA 3D Printer, KINGS, China) with transparent acrylonitrile butadiene styrene (ABS). A fan was purchased from Taobao. The prepared BCNM with the dimensions of 18 × 6 cm was rolled into a cylindrical filter module. The assembled air purifier was put into a 55 L box for indoor air filtration evaluation in a laboratory of the City University of Hong Kong. NaCl aerosol was infused into the chamber until ≈1000 µg m^−3^ and the air purifier was turned on with a flow rate of 4, 8, and 10 m s^−1^, respectively. The BCNM was Joule‐heated at 80 °C. The background concentration in the chamber and the natural decay process without the air purifier were also measured. All particle concentration measurements were done using an A4‐CG laser sensor. The microbial proliferation after 12 h on the basal agar cultured in the box with and without opening the air purifier was monitored. The operation of the electrothermal filter and fan was powered by a lithium battery (150 000 mAh, 300 W, Xinpink, China) charged using a 24 W photovoltaic module (14.32 VDC, 2.32 A, Xingpuguang, China) interfaced with a solar charge controller (20 A, PWM, Xingpuguang, China). The wind velocity of the fan and the inactivation temperature of the BCNM filter were individually controlled by the adjustable DC transformer (SY‐DC300P, Yuanshi, China).

### Characterization

Fourier transform infrared (FTIR) spectrum was conducted using an FTIR Spectrometer (Nicolet iS10, Thermo Scientific, USA). X‐ray photoelectron spectra (XPS) were performed on a scientific ESCALAB 250 instrument. The morphology characteristics of BCNM were captured using scanning electron microscopy (SEM, FEI Quanta FEG 450, Japan) and transmission electron microscopy (TEM, FEI Talos F200X G2, Thermo Scientific, USA) with the mapping function. An image processing software (ImageJ) measured the fiber diameter and pore size of all membranes by processing SEM images. The porous structure was also analyzed by Brunauer–Emmette–Teller (BET) method (ASAP 2460, Micromeritics, USA) and the mercury intrusion method (AutoPore IV 9500, Micromeritics, USA). The mechanical property was measured by a tensile testing machine (ZQ‐990B, Zhiqu, China). The resistance of samples was measured by Keithley 2400 (Tektronix, USA) with a four‐point probe (HP‐504, 4Probes Tech, China) for electrical conductivity calculation. The thermal property, thermal conductivity, and specific heat capacity of BCNM were detected by a thermogravimetric analyzer (TGA 5500, TA, USA), a hot disk (TPS 2500 S, Hot Disk AB, Sweden), and a differential scanning calorimetry (DSC 3, Mettler, Sweden). The zeta potential of samples was measured by an electrokinetic analyzer (SurPASS 3, Anton Paar, Austria).

### Statistical Analysis

All quantitative data were statistically analyzed and fitted using OriginPro 2021 unless otherwise stated. The error bars presented in the figures represent the standard deviation (SD) of three independent measurements (*n* = 3). Statistical variations were analyzed to ensure data reproducibility, and detailed conditions for each measurement are described in the respective figure captions.

## Conflict of Interest

The authors declare no conflict of interest.

## Supporting information



Supporting Information

Supplemental Video 1

Supplemental Video 2

Supplemental Video 3

## Data Availability

The data that support the findings of this study are available from the corresponding author upon reasonable request.
