# Supplementary material for: Bridged Conductive Nanofibrous Membrane Overcoming the Porosity‐Conductivity Trade‐Off for Electrothermal Air Purification
Source: Adv Sci (Weinh). 2025 Nov 6;12(47):e08650. doi: 10.1002/advs.202508650 (PMC12713030; doi:10.1002/advs.202508650)
Supplement: Supplementary file 1 — Supporting Information [file ADVS-12-e08650-s001.docx]

**Bridged Conductive Nanofibrous Membrane Overcoming the Porosity-Conductivity Trade-Off for Electrothermal Air Purification**

*Xiaoxue Yao^a#*^, Zhenwen Zhang^a#^, Wei Deng^a^, Chuhan Feng^b^, Qili Xu^a^, Wenzhu Lin^c^, Zehua Peng^d^, Yang Cao^e^, Wang Guo^b^, Dr. Bee Luan Khoo^b*^, Prof. Steven Wang^a*^*

*^a^ Department of Mechanical Engineering, City University of Hong Kong, 83 Tat Chee Avenue, Kowloon, Hong Kong, 999077, China*

*^b^ Department of Biomedical Engineering, City University of Hong Kong, 83 Tat Chee Avenue, Kowloon, Hong Kong, 999077, China*

*^c^ School of Materials and Energy, Guangdong University of Technology, Guangzhou 510006, China*

*^d^ Department of Mechanical Engineering, The Hong Kong Polytechnic University, Hung Hom, Kowloon, Hong Kong, 999077, China*

*^e^ Department of Mechanical Engineering, The University of Hong Kong, Pokfulam Road, Hong Kong, 999077, China*

# These authors contribute equally to this work

Corresponding author: [xiaoxuyao3@cityu.edu.hk](mailto:xiaoxuyao3@cityu.edu.hk); [blkhoo@cityu.edu.hk](mailto:blkhoo@cityu.edu.hk); [steven.wang@cityu.edu.hk](mailto:steven.wang@cityu.edu.hk)

**Figure S1.** FTIR spectra of pure PAN membrane and BCNM.


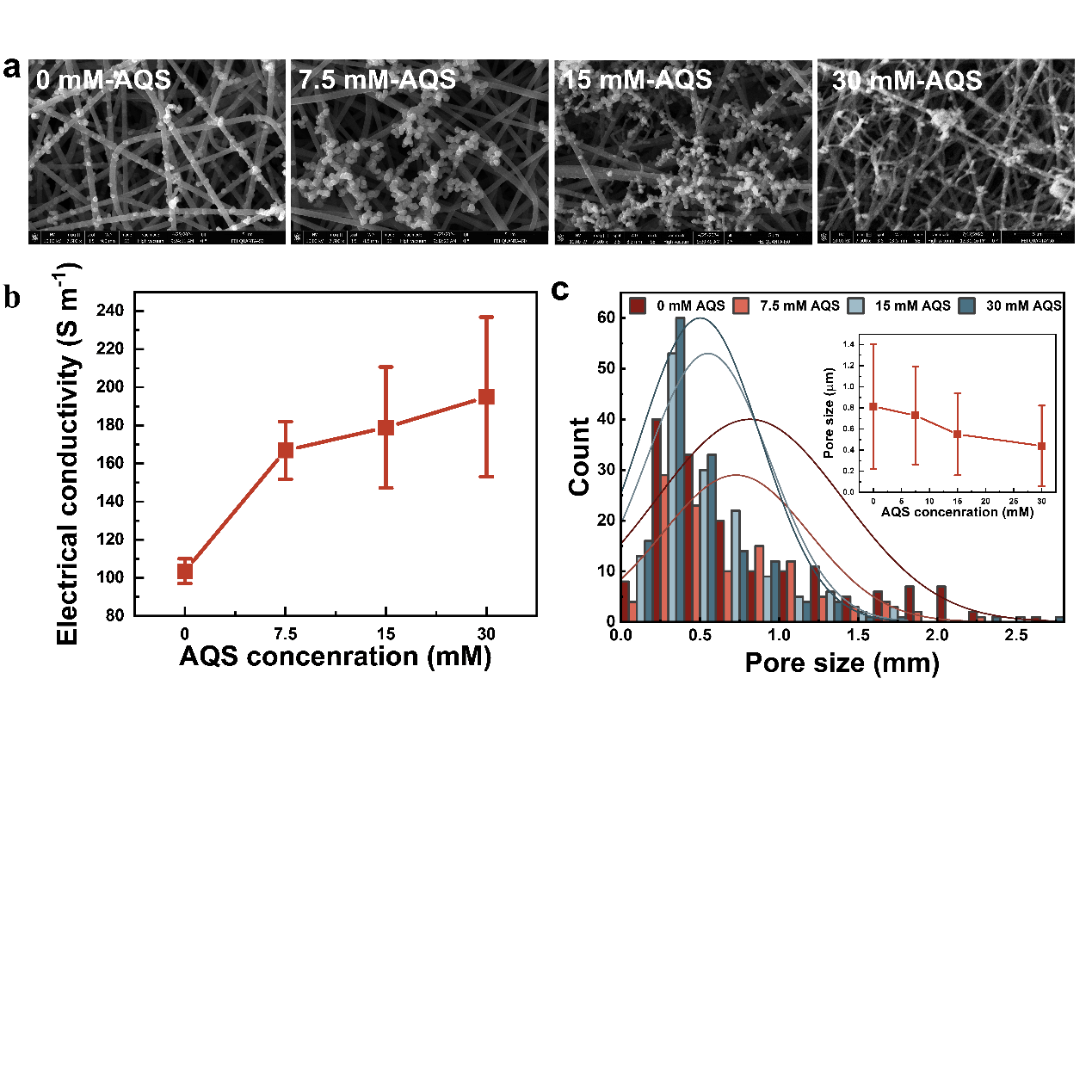


**Figure S2.** AQS surfactant concentration impact on BCNM morphology and conductivity. (a) Morphology characterization of BCNMs fabricated by gradient concentration of AQS dopants and (b) their electrical conductivity as well as (c) their pore size histograms with average pore sizes in the inset.


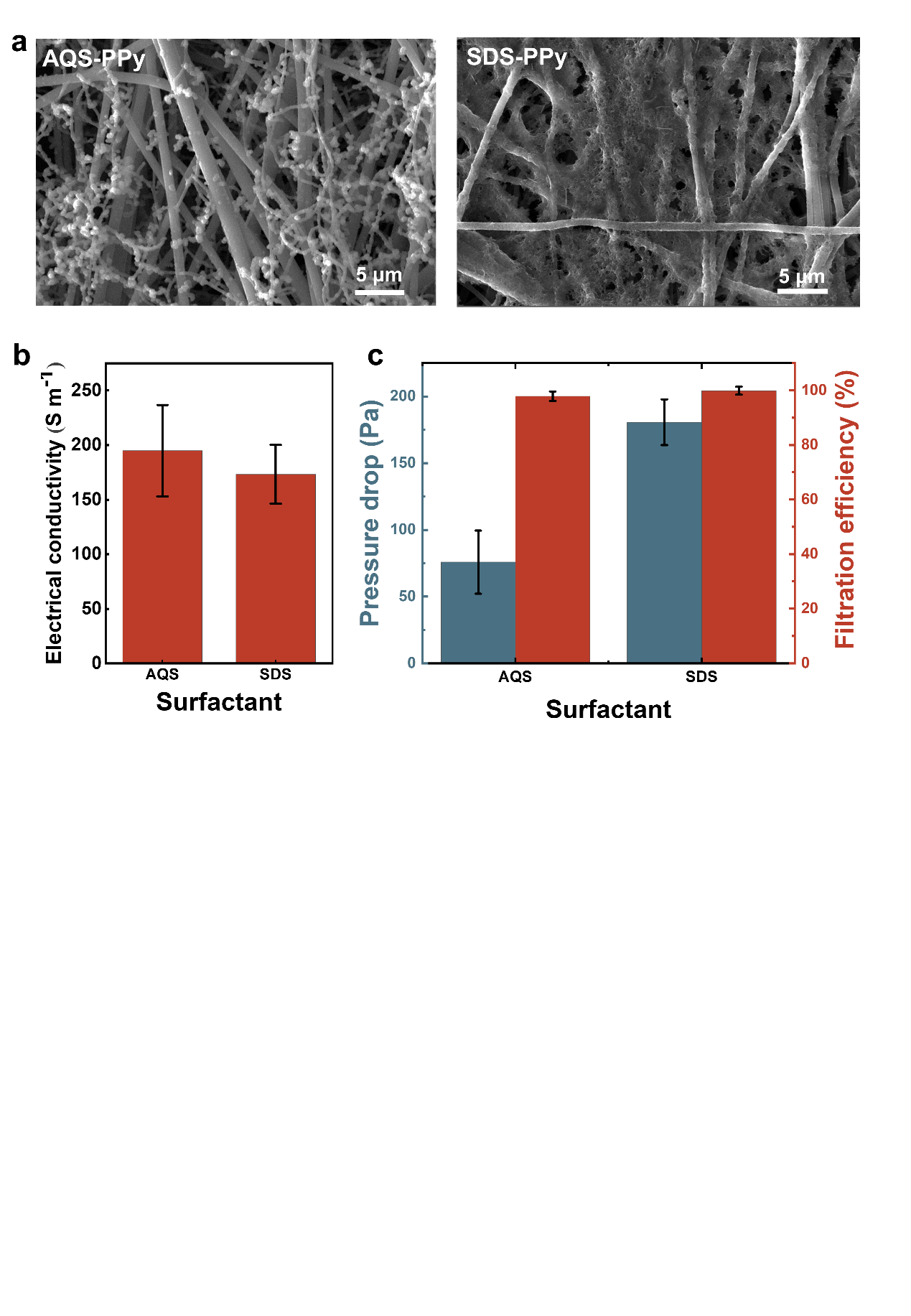


**Figure S3.** Impact of dopant type on PPy synthesis and membrane performance. (a) SEM images of PAN@PPy membranes synthesized by AQS and SDS as dopants. (b) Electrical conductivity of AQS- and SDS-coated PAN@PPy membranes. (c) Pressure drop and filtration efficiency of AQS- and SDS-coated PAN@PPy membranes.


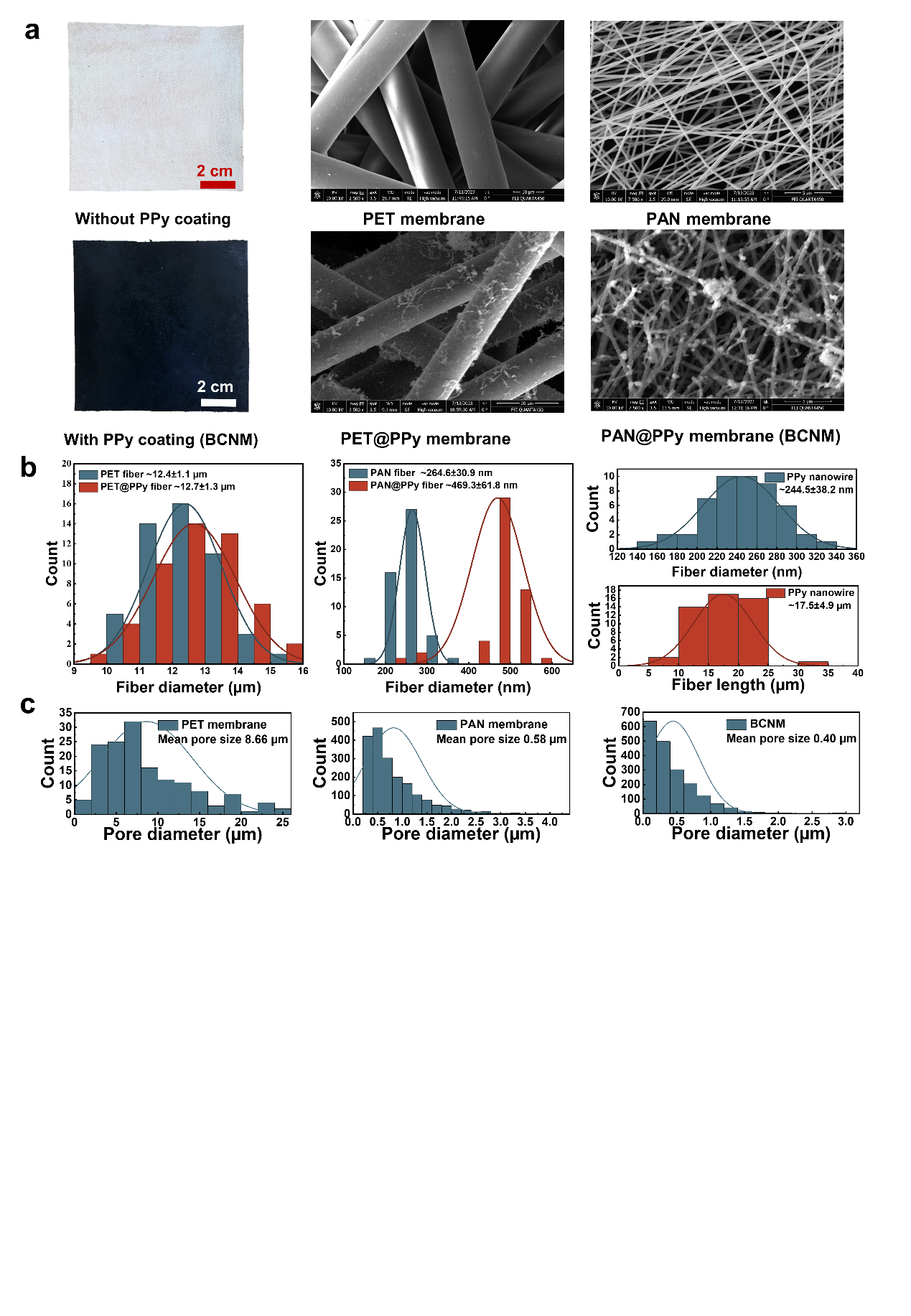


**Figure S4.** Morphology characterization of BCNM. (a) Picture and SEM images of BCNM before and after PPy polymerization. (b) Fiber diameter histograms of BCNM before and after PPy polymerization. (c) Pore diameter histograms of PET substrates, PAN membrane, and BCNM.


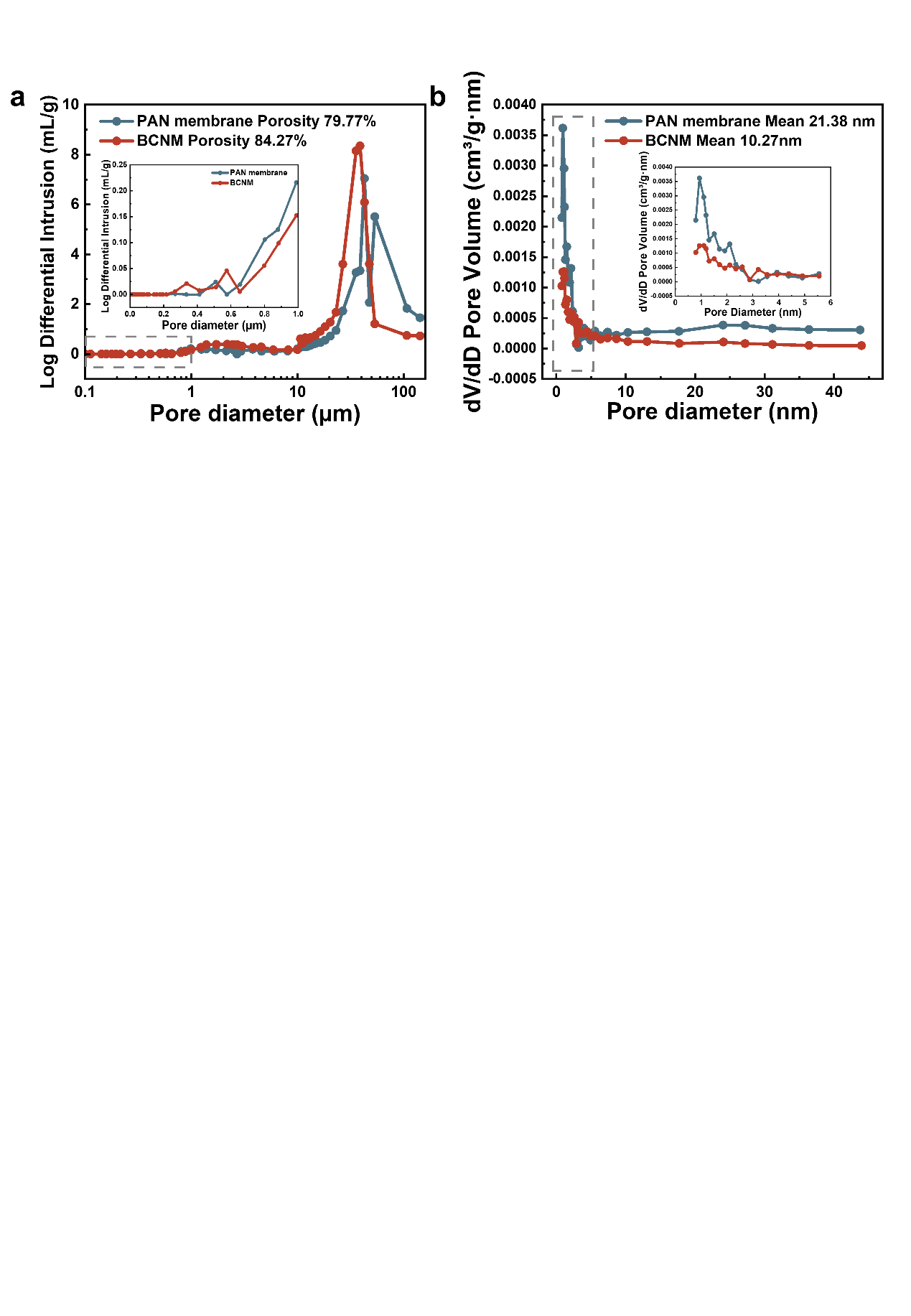


**Figure S5.** Pore structure characterization. (a) Pore size distribution of PAN membrane and BCNM measured by Mercury intrusion porosimetry. (b) Pore size distribution of PAN membrane and BCNM measured by BET.


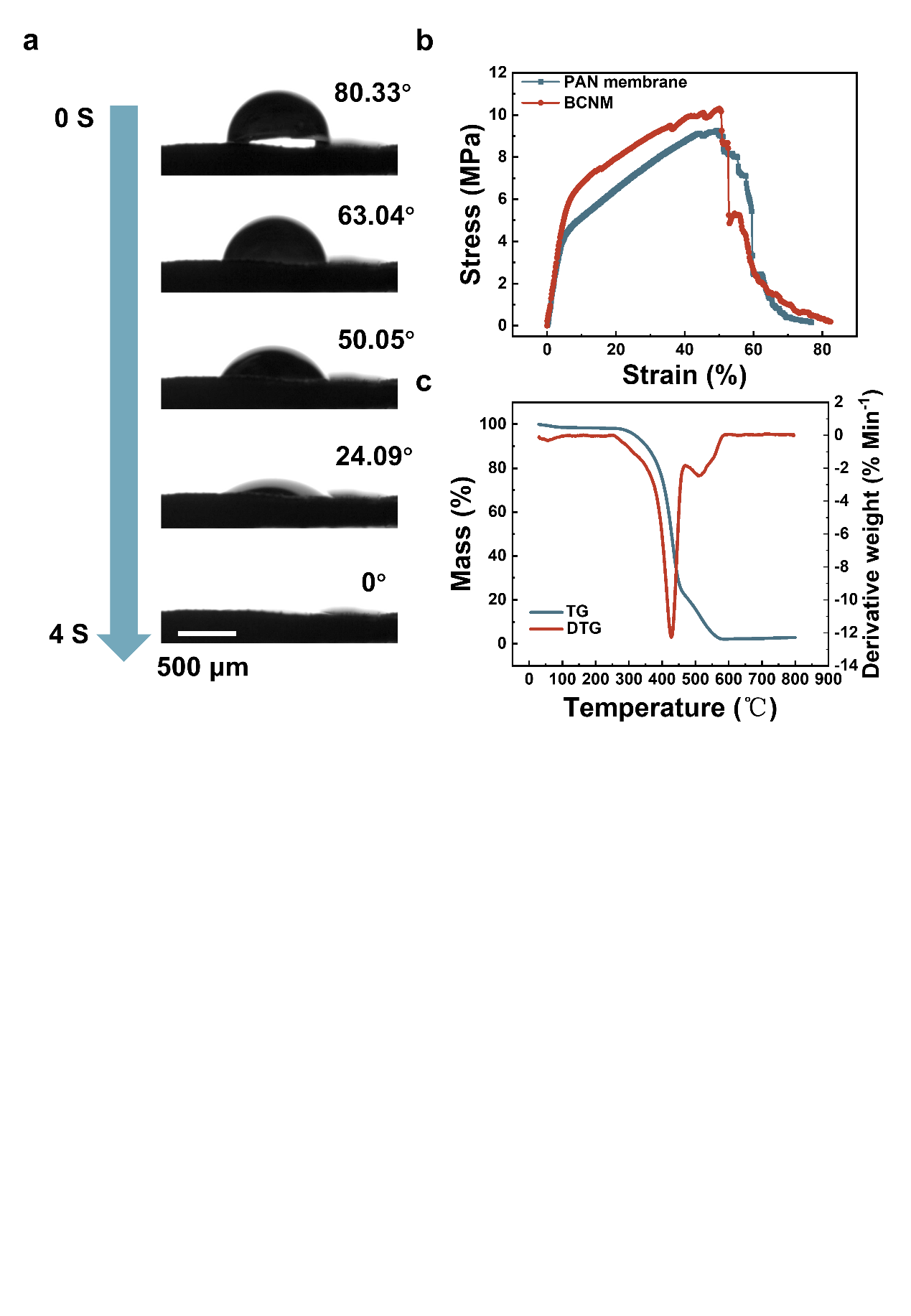


**Figure S6.** Property characterization of BCNM. (a) Superhydrophilicity. (b) Stress-strain curves before and after PPy coating. (c) TG and DTG curves.


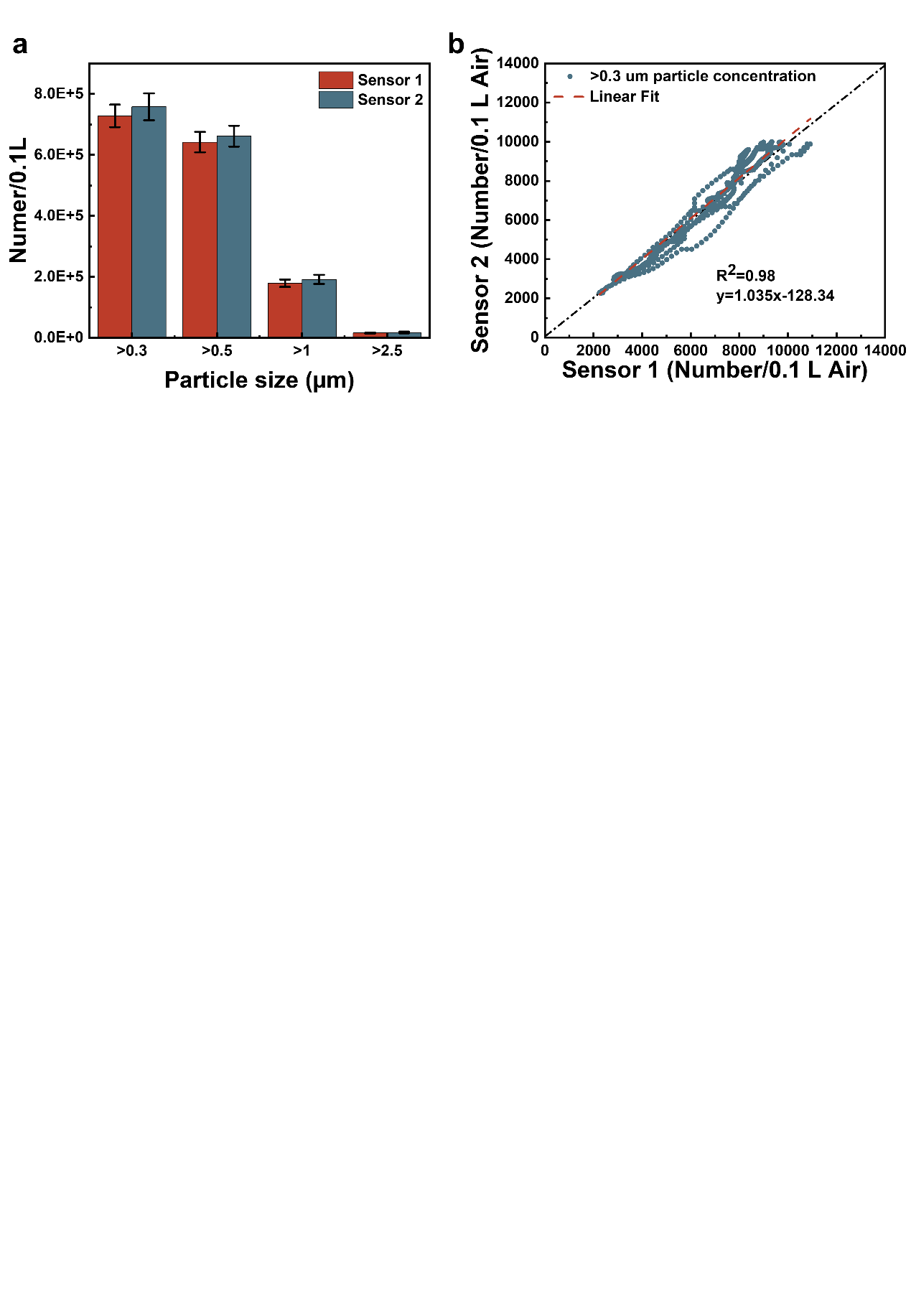


**Figure S7.** Calibration of upstream and downstream particle counting sensors.


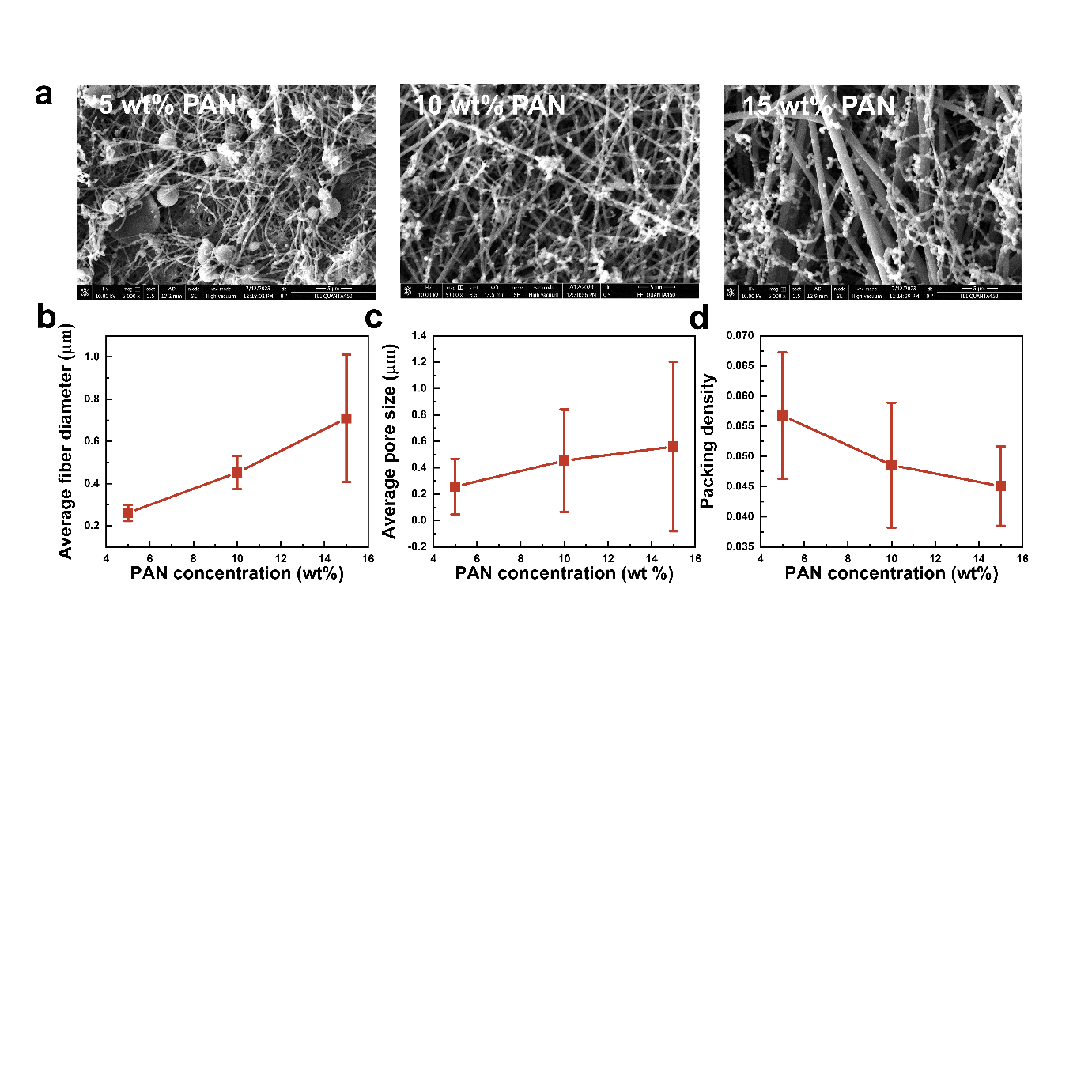


**Figure S8.** Characterization of BCNMs fabricated by different PAN concentrations. (a) SEM images of BCNMs fabricated by 5 wt% PAN, 10 wt% PAN, and 15 wt% PAN. (b) Average fiber diameter, (c) Average pore size, and (d) Packing density of these BCNMs.


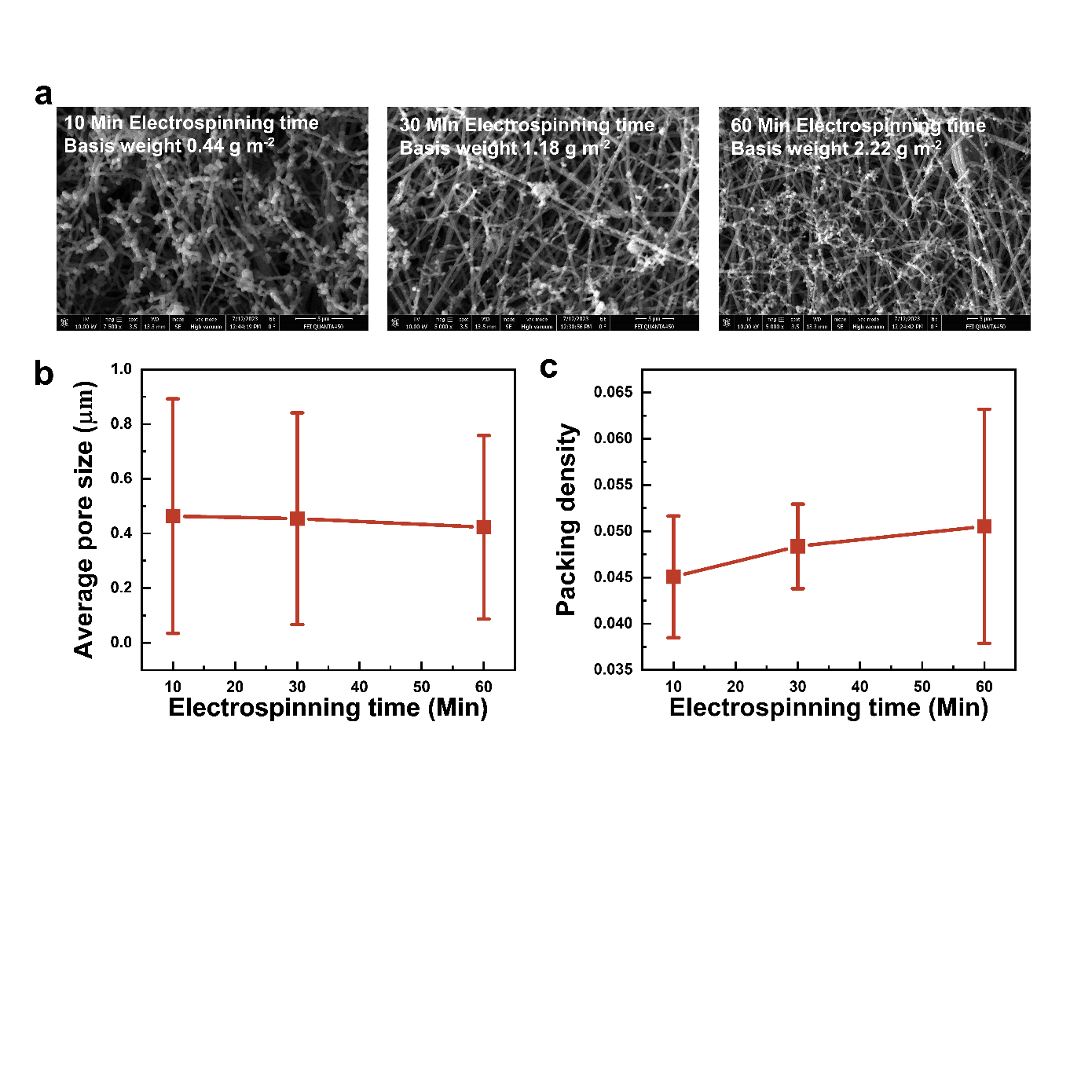


**Figure S9.** Characterization of BCNMs fabricated by different electrospinning times. (a) SEM images of BCNMs fabricated by 10 wt% PAN under 10 Min, 30 Min, and 60 Min. (b) Average pore size and (c) Packing density of these BCNMs.


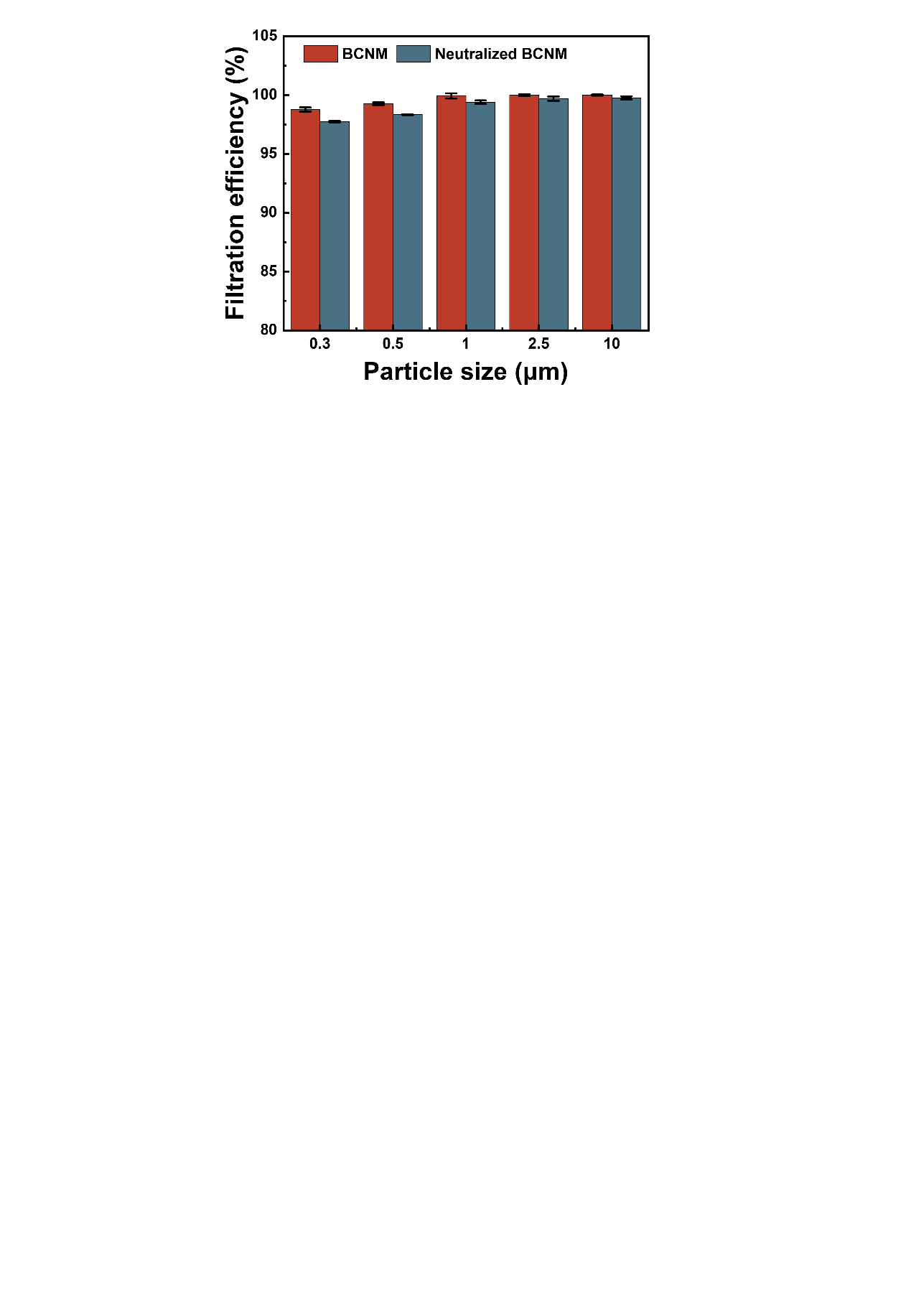


**Figure S10.** Filtration efficiency of BCNM before and after surface charge neutralization.


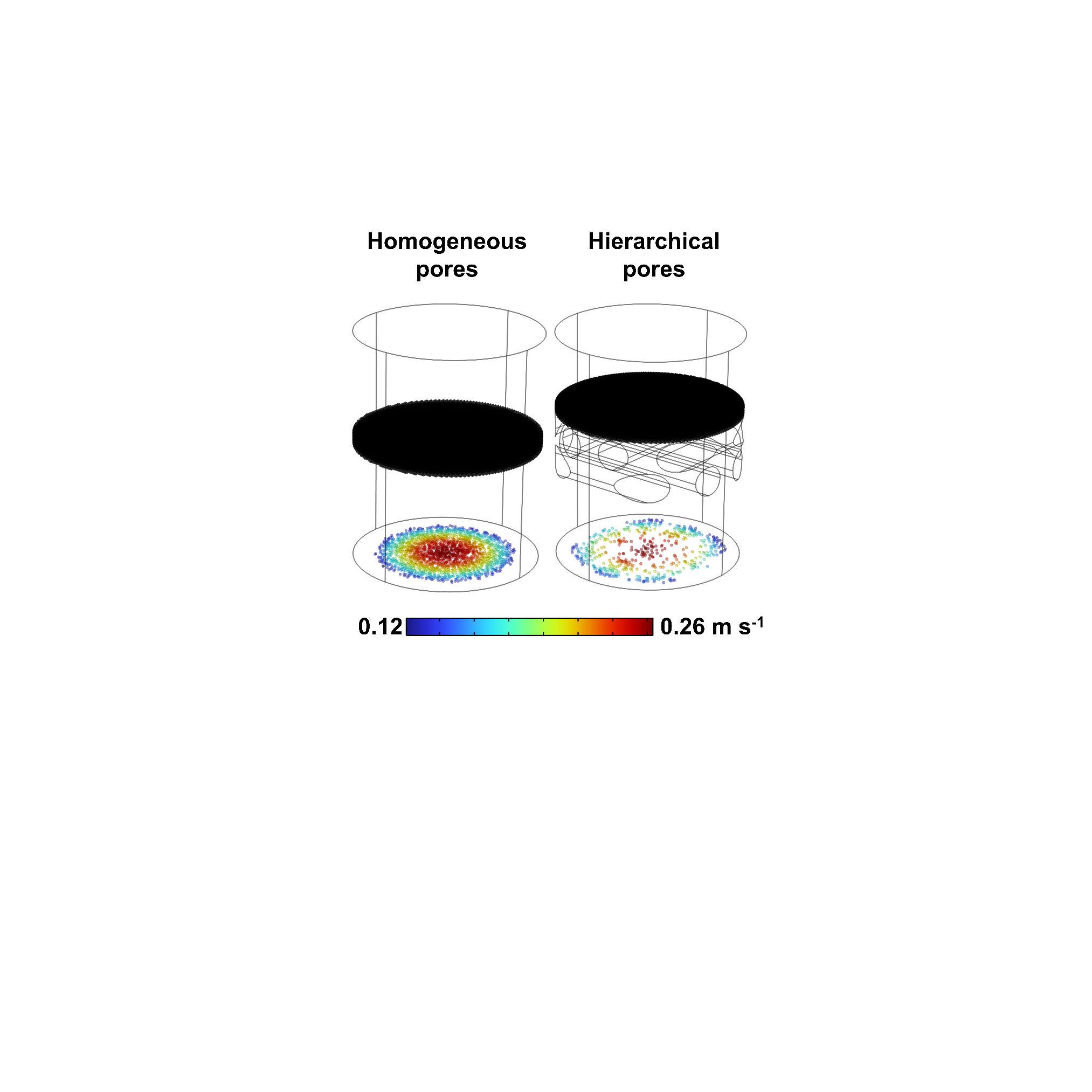


**Figure S11.** Simulation comparison of filtration efficiency between the homogeneous fibrous model and hierarchical fibrous model based on the experimental data.


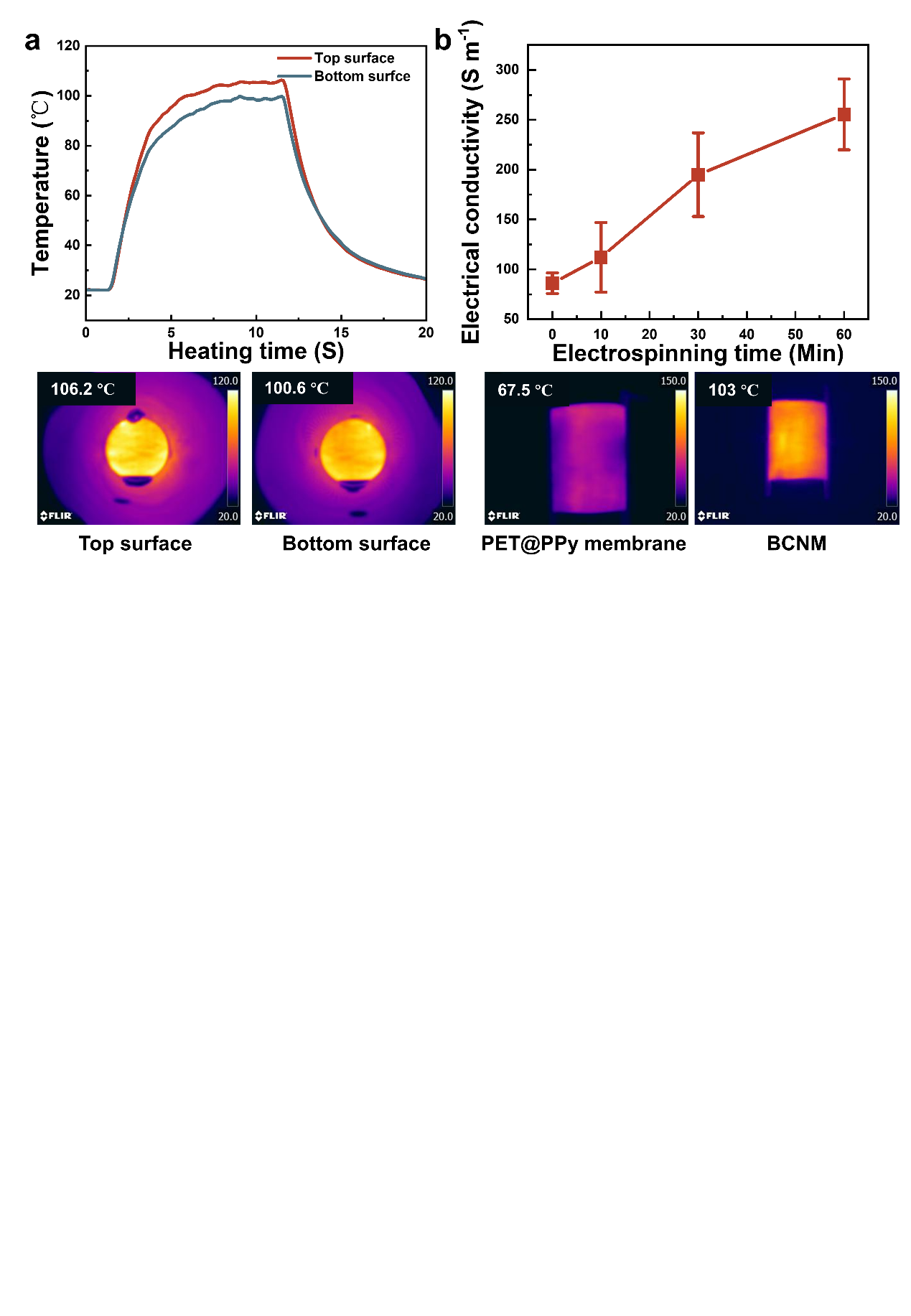


**Figure S12.** Electrothermal performance of Joule‐heated BCNM. (a) Thermocouple and infrared temperature measurements of the front and back surfaces of BCNM. (b) Effect of electrospinning parameters on the electrical conductivity of BCNM, and comparison of infrared temperatures between PET@PPy membrane and BCNM.

**
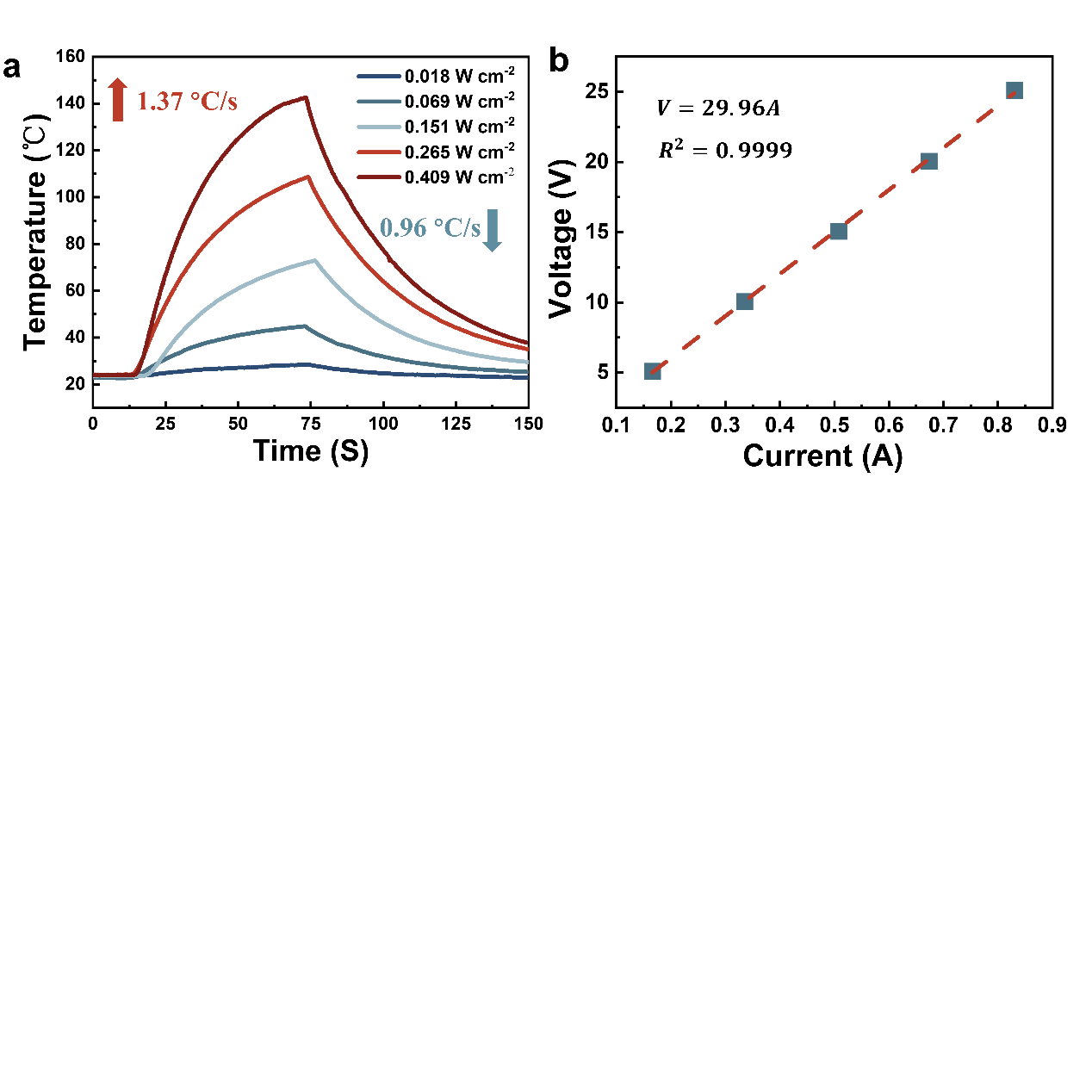
Figure S13.** Electrothermal properties. (a) Surface temperature change curves with time of a PI-Metal heater powered by gradient power density. (b) Voltage and current relationship of BCNM.


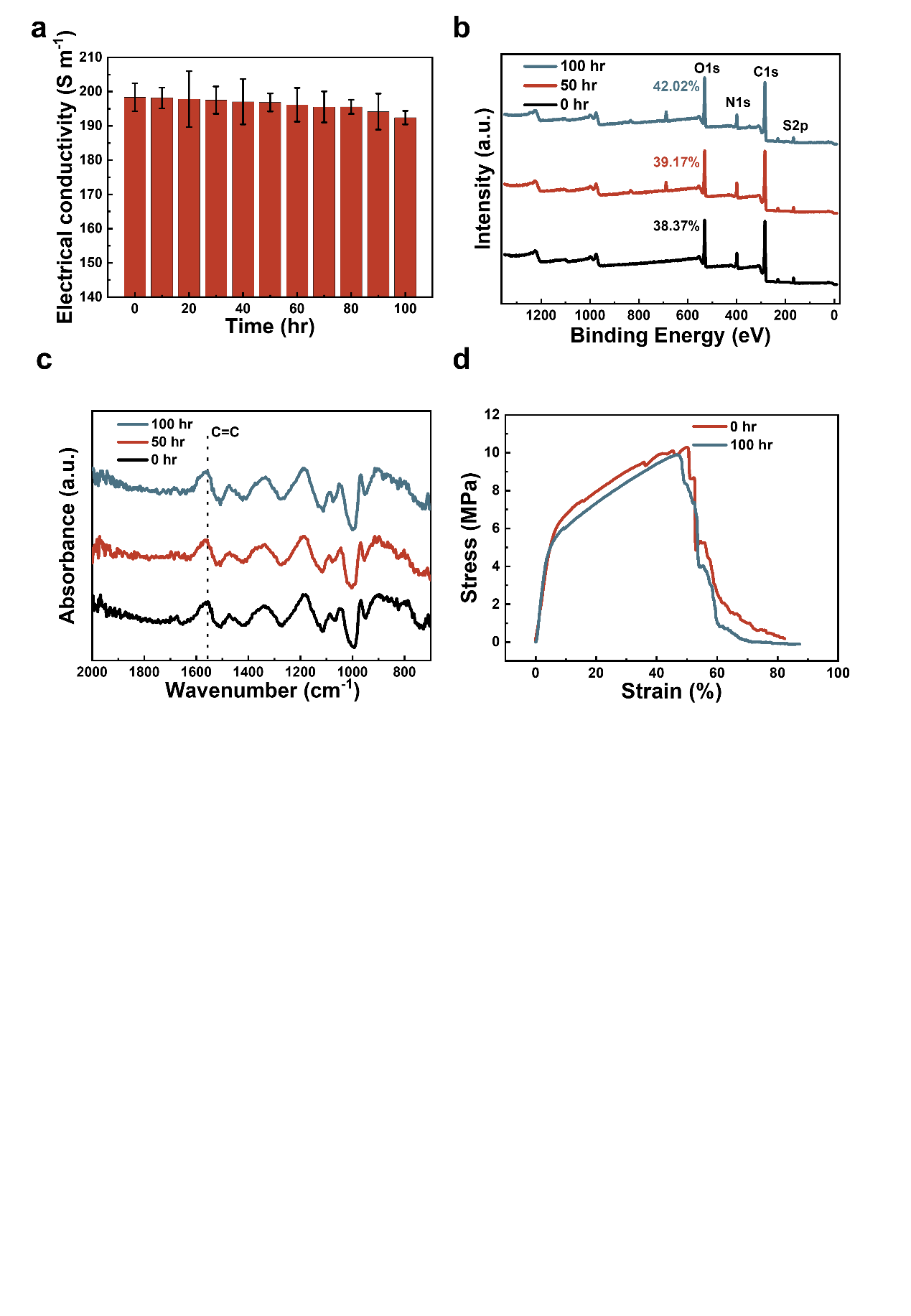


**Figure S14.** Aging performance characterization of BCNM. (a) Electrical conductivity. (b) XPS spectra. (c) FTIR spectra. (d) Strain-stress curves.


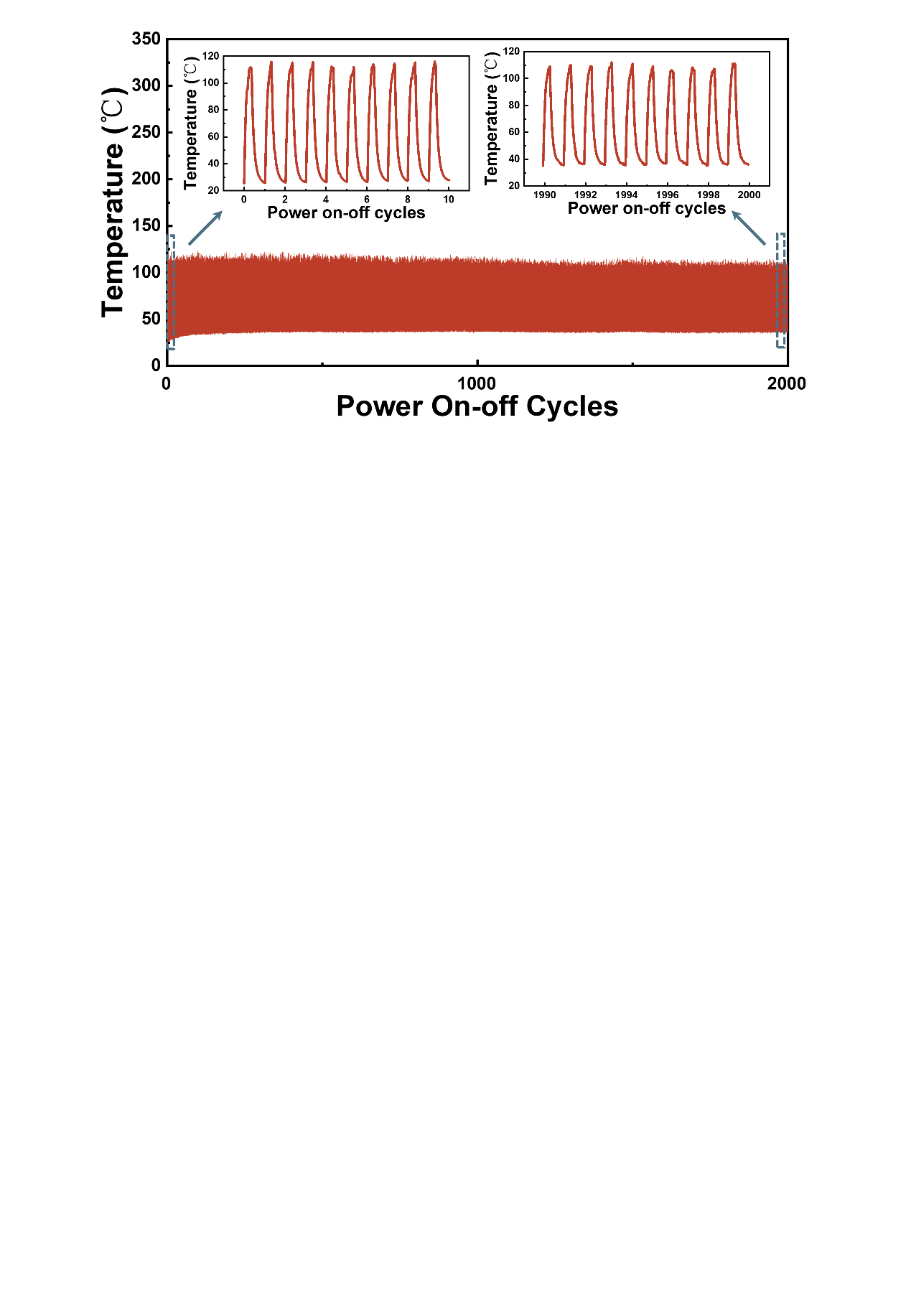


**Figure S15.** Electrothermal stability of BCNM during 2000 on-off power cycling tests (power density of 0.17 W cm^-2^).


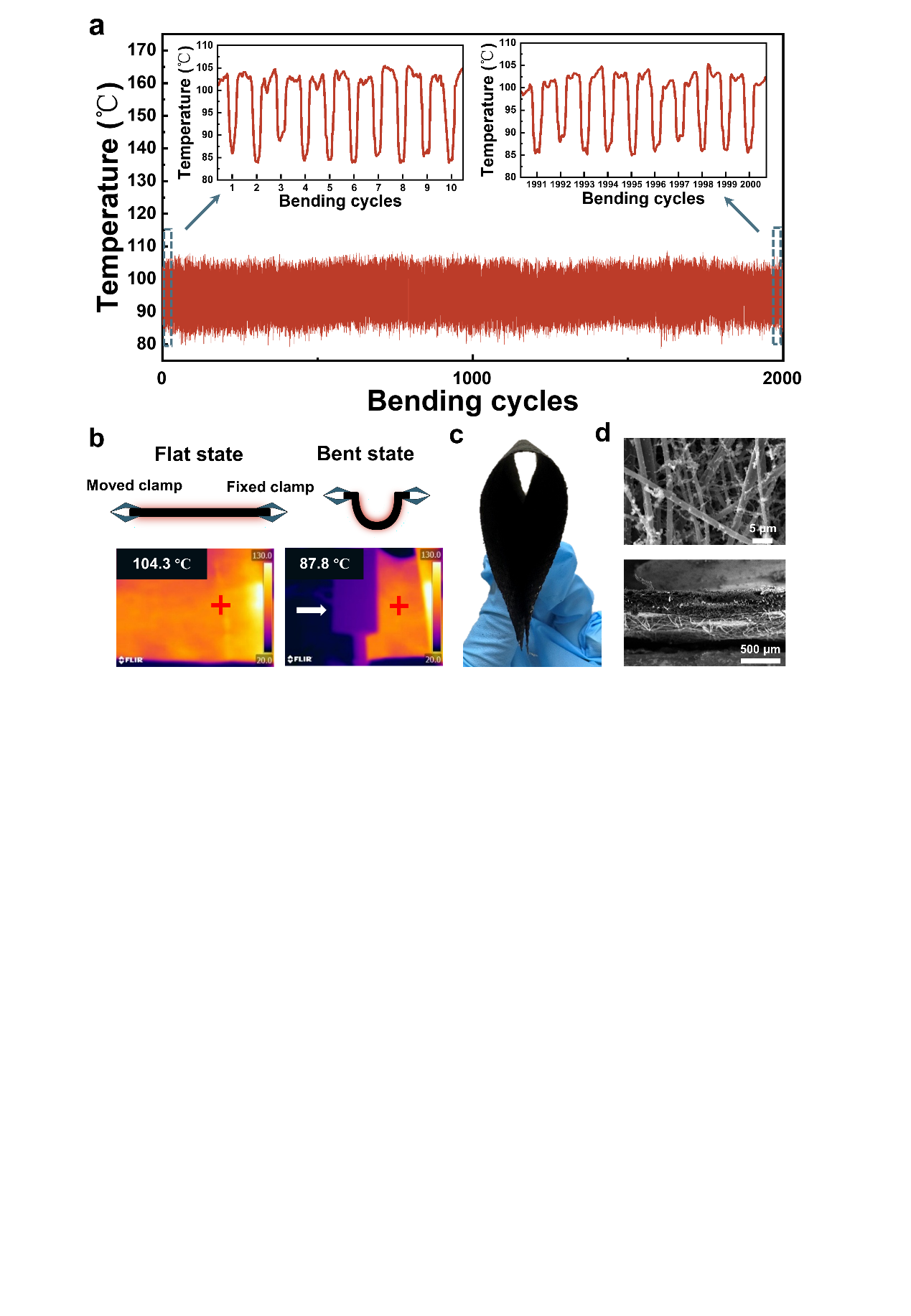


**Figure S16.** Electrothermal stability of BCNM under mechanical deformation. (a) Temperature variation of BCNM during 2000 bending cycles (power density of 0.13 W cm^-2^). (b) Schematic illustration of the bending test and corresponding infrared thermal images. (c) Photograph of BCNM after bending cycles. (d) SEM images of the top and side surfaces at the bending region.


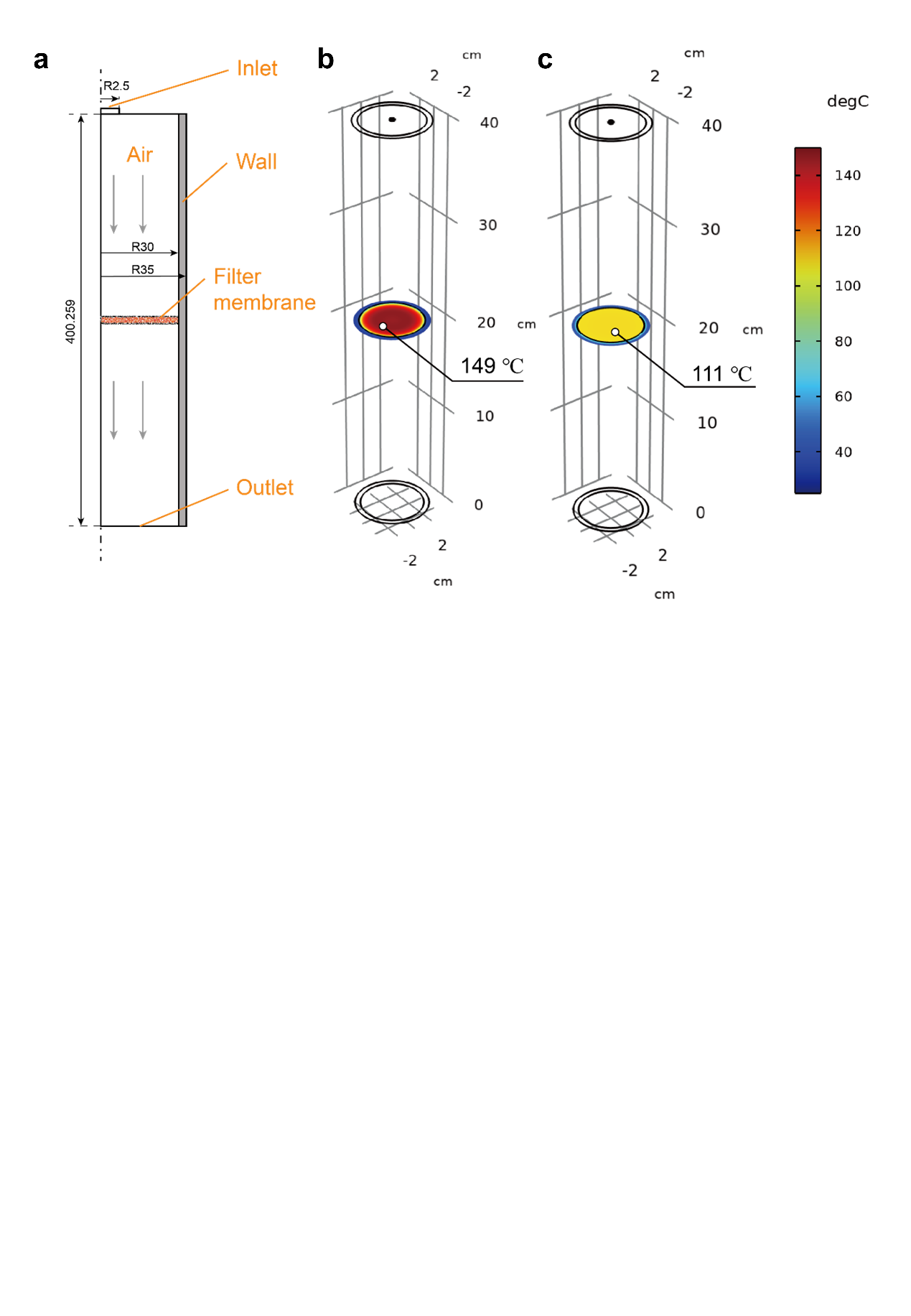


**Figure S17.** Temperature comparison of filter membranes with high and low thermal conductivity. (a) Geometry of numerical simulation. (b) Thermal distribution of low thermal conductivity BCNM membrane (κ=0.08 W m^-1^ K^-1^). (c) Thermal distribution of high thermal conductivity membrane (κ=2000 W m^-1^ K^-1^).


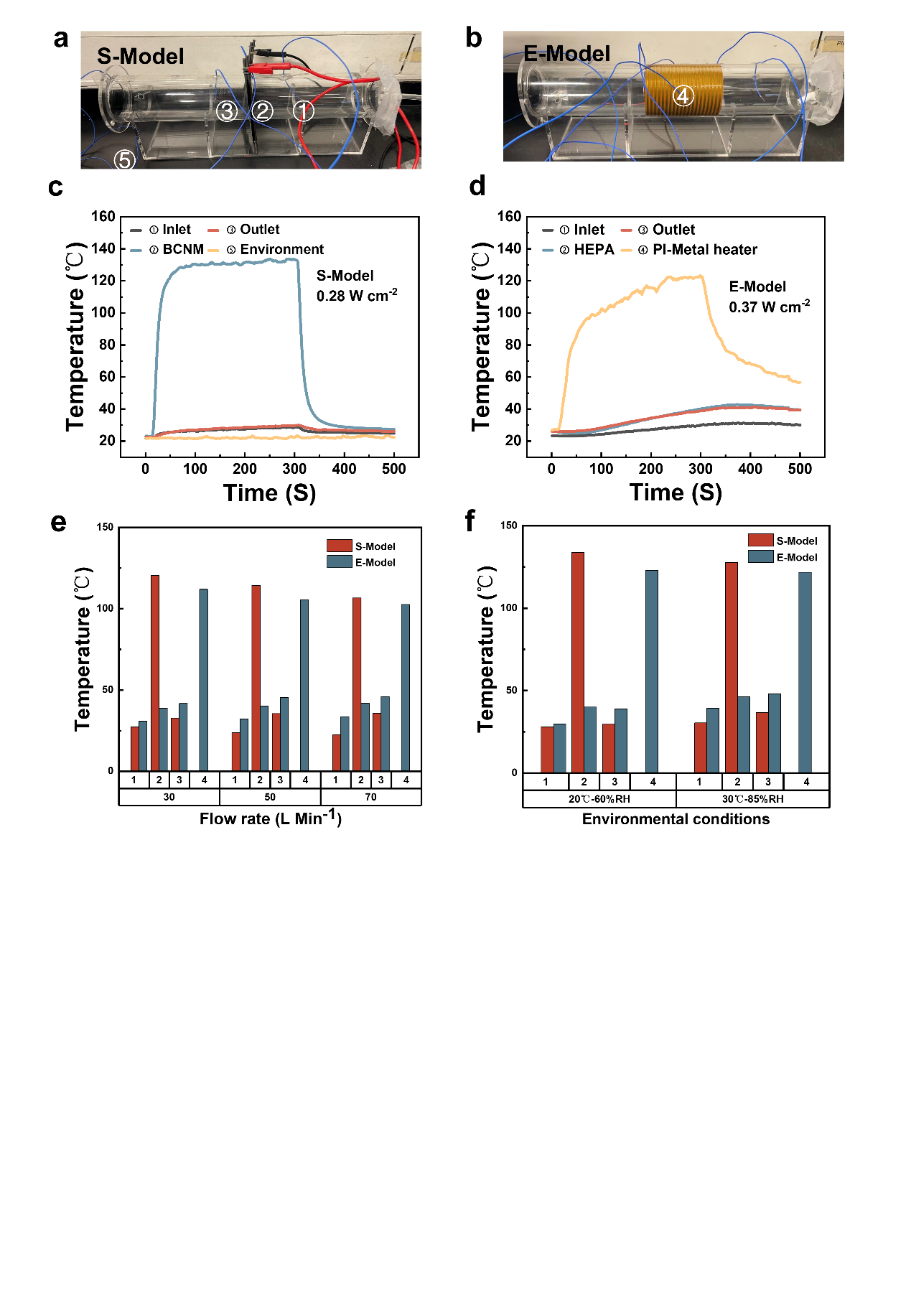


**Figure S18.** Thermal distribution comparison between the S-Model and E-Model. Photographs of (a) BCNM and (b) HEPA filter mounted in the ventilation tubes. Dynamic heating and cooling curves of (c) S-Model and (d) E-Model. (e) Temperature distributions of both models under different airflow rates. (f) Temperature distributions of both models under different ambient temperature and humidity conditions.


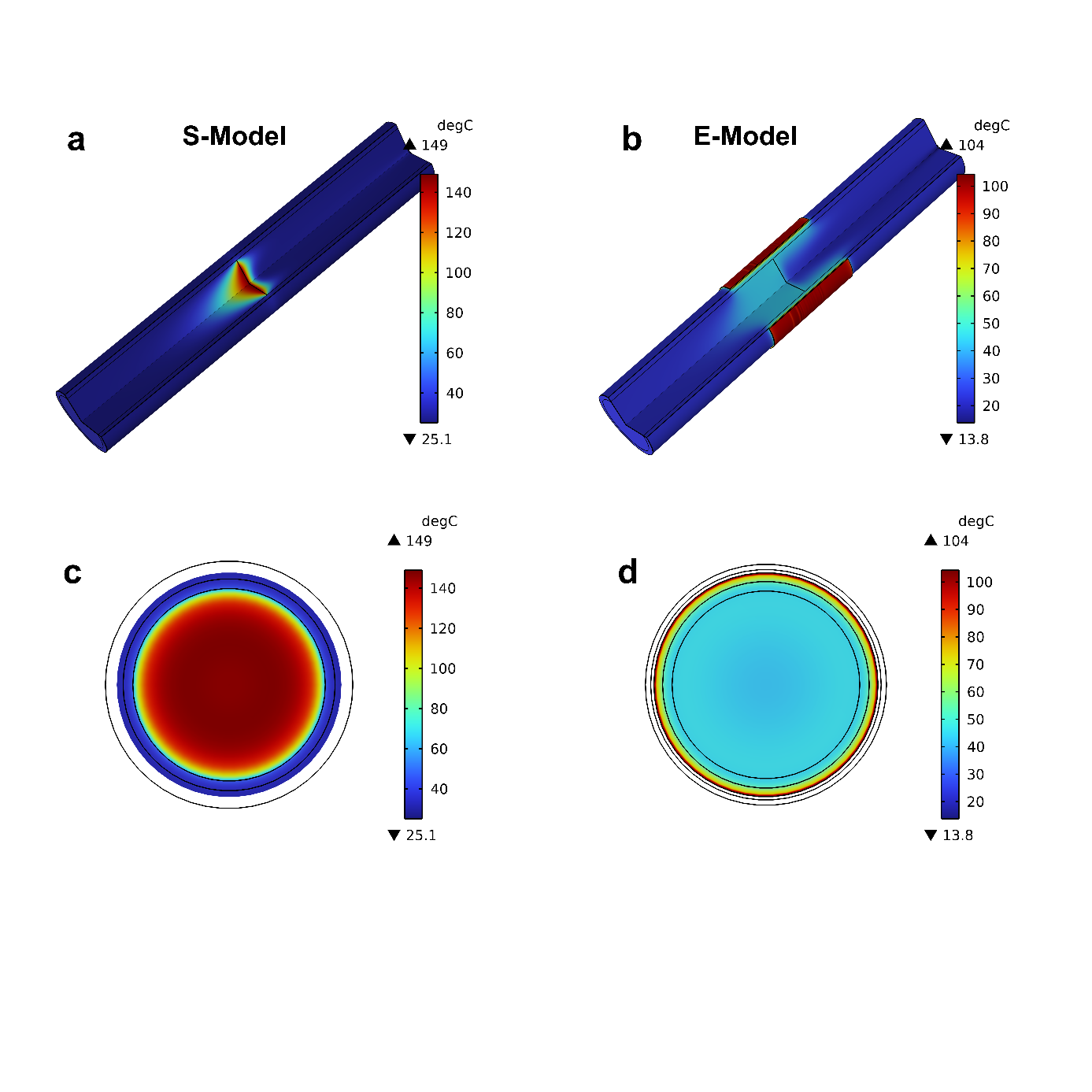


**Figure S19.** The 3D simulation images of the temperature field of (a) S-model and (b) E-model with the temperature profiles of (c) BCNM and (d) HEPA in these two models.


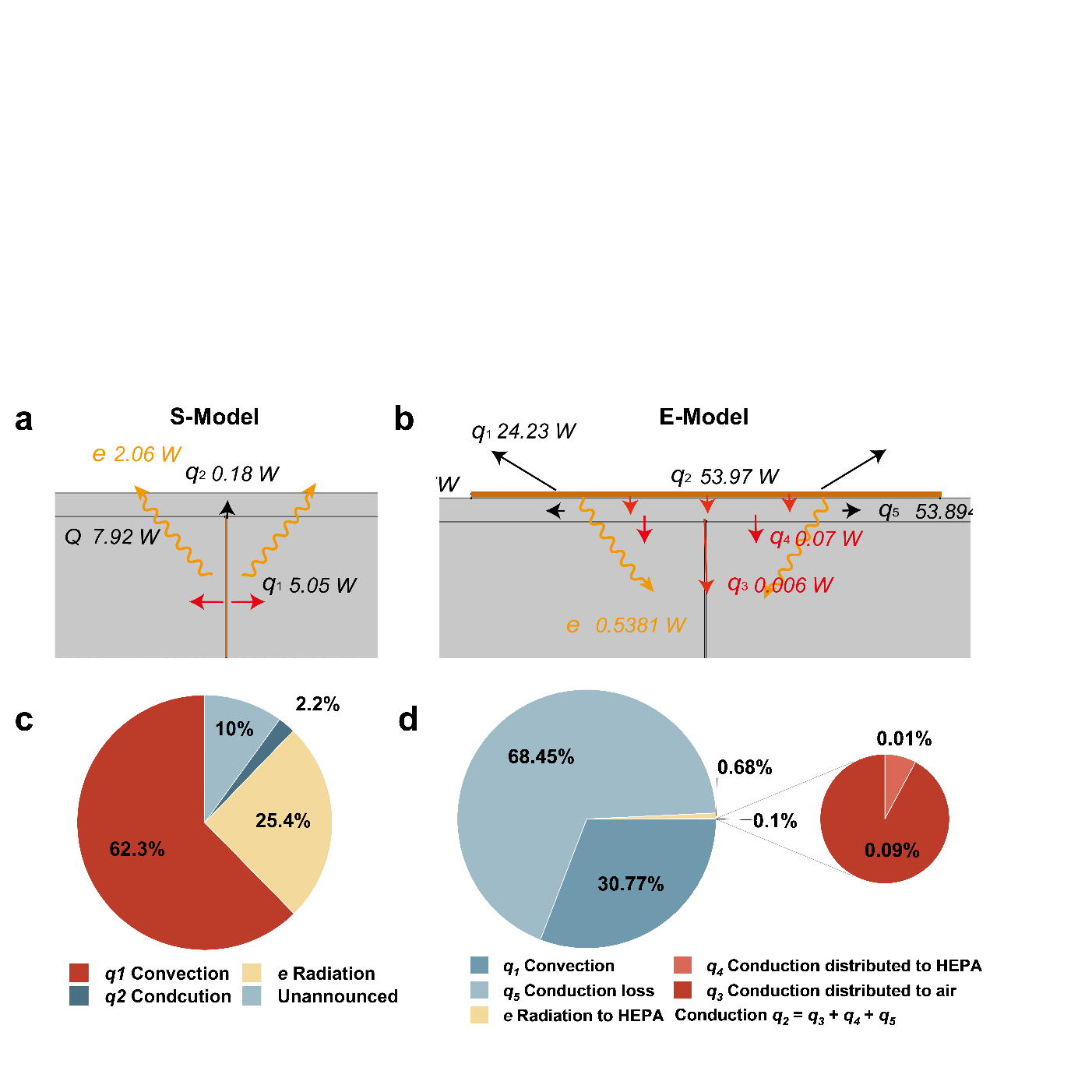


**Figure S20.** Energy balance diagrams of (a) S-model and (b) E-model. Pie charts for the contribution of each factor to the overall energy consumption of (c) S-model and (d) E-model by simulation and calculation.


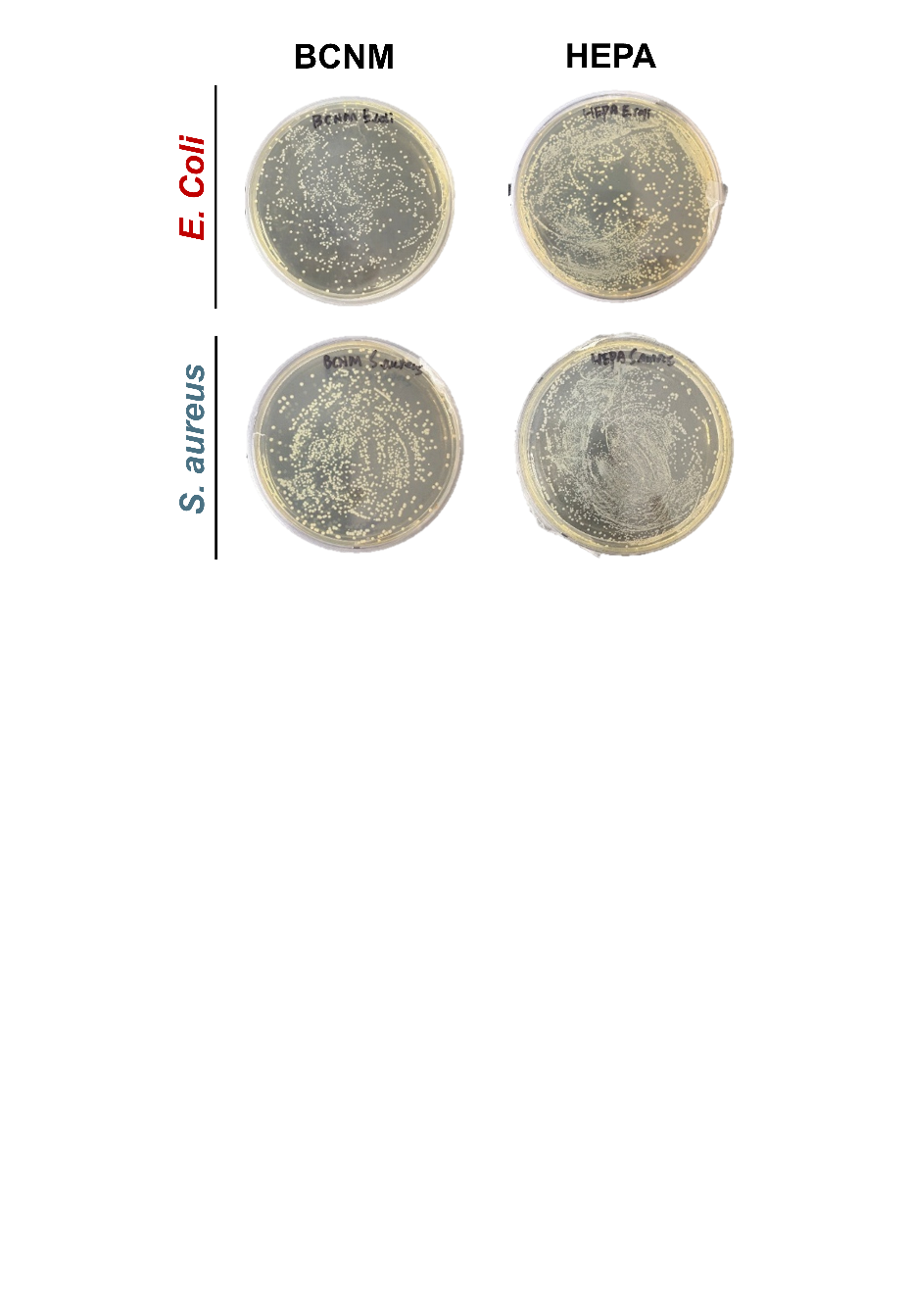


**Figure S21.** Colony distribution on agar plates of the non-thermal antibacterial test.


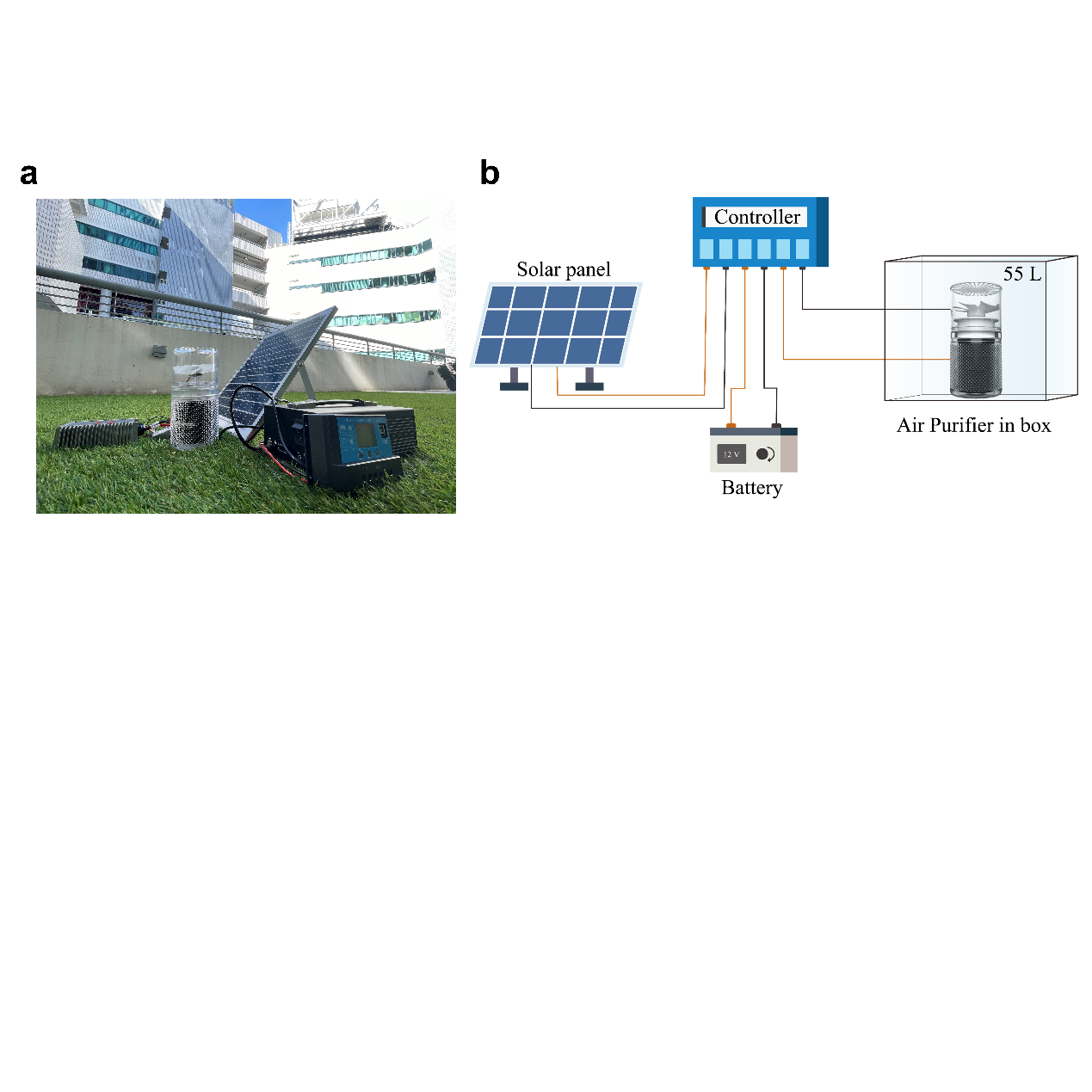


**Figure S22.** (a) photograph and (b) schematic illustration of the BCNM-based air purifier system.


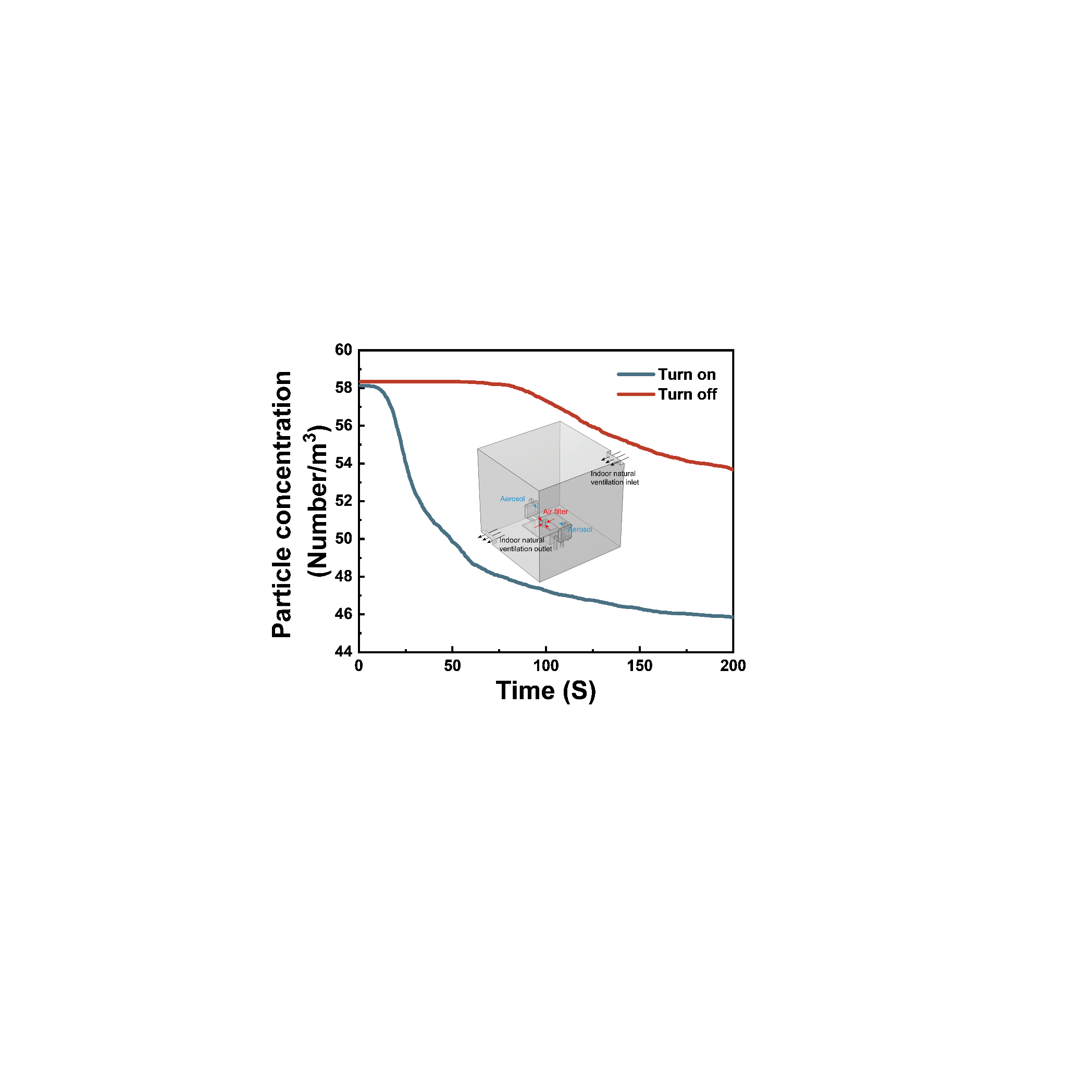


**Figure S23.** Simulation data of particle concentration distribution with time in a 125 m^3^ room when a BCNM-purifier turns on and off.

**Table S1** Performance comparison of BCNM with previously reported air filters

| Air filters | | Pressure drop (Pa) | Filtration efficiency (%) | QF  (Pa-1) | Test flow rate | Test particle size (μm) | Test particle type | Reference |
| --- | --- | --- | --- | --- | --- | --- | --- | --- |
| Electrospun hybrid filters | PAN/ZIF-67 | 100 | 0.7 | 0.012 | 15 cm/s | 0.3-0.4 | NaCl aerosol | [1] |
|  | PAN/TiO2/Ag | 90 | 0.84 | 0.020 | 10 cm/s | 0.3-0.5 | NaCl aerosol | [2] |
|  | PAN/GO/PI-6 | 92 | 0.995 | 0.057 | 20 cm/s | 2.5 | NaCl aerosol | [3] |
|  | PAN/PTHP | 154 | 0.969 | 0.022 | 85 L/min | 0.24 | NaCl aerosol | [4] |
|  | ZIF-8-on-PLA/PHBR | 70 | 0.95 | 0.043 | 11 cm/s | 0.1 | PS particles | [5] |
|  | VIO-PAN NF | 79 | 0.878 | 0.028 | 32 L/min | 0.8-3.4 | NaCl aerosol | [6] |
|  | PAN/Ni-CAT-1 | 75 | 0.9 | 0.031 | 10 cm/s | 2.5 | Incense sticks | [7] |
|  | PAN/β-CD ENMs | 150 | 0.85 | 0.013 | 9 cm/s | 0.3 | KCl particles | [8] |
| Electrospun multilayer filters | PAN transparent air filter | 133 | 0.96 | 0.024 | 21 cm/s | 2.5 | Incense sticks | [9] |
|  | PEO@PAN/PSU | 95 | 0.99 | 0.099 | 32 L/min | 0.3-0.5 | NaCl aerosol | [10] |
|  | PSU/PAN/PA-6 | 108 | 0.99 | 0.087 | 32 L/min | 0.3 | NaCl aerosol | [11] |
|  | PAN/PAN-FPU | 163.7 | 0.99 | 0.039 | 32 L/min | 0.075 | NaCl aerosol | [12] |
| Electrothermal  filters | Annealing PAN with Cu electroplating | 2750 | 0.93 | 0.001 | 50 L/Min or 43.5 cm/s | 0.50–1.5 | Cu powder | [13] |
|  | CNT aerogel | 1946 | 0.99 | 0.004 | 10 cm/s | 0.006-2.5 | Ag / Dioctyl sebacate aerosol | [14] |
|  | GeFM | 323 | 0.76 | 0.004 | 13 cm/s | 0.3 | Bacillus subtilis and NaCl particles | [15] |
|  | LIG | 300 | 0.86 | 0.007 | 10 cm/s | 0-0.8 | Cigarettes | [16] |
|  | Copper NWs | 85 | 0.68 | 0.014 | 10 cm/s | 0.01-10 | KCl particles and smokes | [17] |
|  | PAN NF/CNT | 132 | 0.99 | 0.049 | 14.16 cm/s | ≤0.3 | NaCl aerosol | [18] |
|  | This work | 76 | 0.987 | 0.058 | 30 L/Min (13 cm/s) | >0.3-10 | Charged NaCl aerosol | - |
|  |  | 43 | 0.985 | 0.098 | 32 L/Min  (5.33 cm/s) | ≤0.3 | Neutralized NaCl aerosol | - |

*The comparison includes data from studies using different aerosol types and testing conditions. As such, the performance values are not directly comparable and are presented for general reference only.

**Table S2** Cost calculation of BCNMs

| Raw material | Specification | | Price ($) | Usage per BCNM | Unit Price ($) | |
| --- | --- | --- | --- | --- | --- | --- |
| PET fibers | 1.6 m x 0.5 m | | 1.39 | 10 cm x 10 cm | | 0.017 |
| PAN | 500 g | | 199.77 | 0.1 g | | 0.04 |
| Py | 100 g | | 31.87 | 0.6 g | | 0.191 |
| AQS | 25 g | | 14.26 | 0.5 g | | 0.285 |
| SSA | 100 | | 5.94 | 5 g | | 0.297 |
| FeNO_3_ | 500 g | | 5.81 | 8 g | | 0.093 |
| DMF | 500 g | | 7.74 | 0.9 g | | 0.014 |
|  |  |  | | Total price | | 0.937 |

*The estimated cost of BCNM represents raw material expenses only (based on Aladdin prices) and excludes factors for commercial-scale production such as labor, energy consumption, and waste management.

**Table S3** Comparison of BCNMs with commercial air purification technologies

| Air filtration technology | HEPA-UVGI | ESP | LIG | BCNM |
| --- | --- | --- | --- | --- |
| Filtration efficiency (%) | 99.97 | 78.60 | 86 | 98.79 |
| Pressure drop (Pa) | 290 | 67 | 300 | 76 |
| QF (Pa^-1^) | 0.028 | 0.023 | 0.007 | 0.058 |
| Antibacterial efficiency (%) | 90.00 | 72.20 | 99.99 | 99.49 |
| Fan power consumption (W) | 2.18 | 2.36 | 0.42 | 0.14 |
| Sterilization power consumption (W) | 45 | 10 | 33 | 13 |
| Total power consumption (W) | 47.18 | 12.36 | 32.95 | 13.14 |
| 1/Power consumption | 0.212 | 0.081 | 0.030 | 0.076 |
| Cost ($) | 79.000 | 10.000 | 513.000 | 0.937 |
| 1/Cost | 0.013 | 0.100 | 0.002 | 1.067 |
| Reference | [^[19,20]^ | [21,22] | [16] | This work |

1. The power consumption (P_t_) in a simplified air filtration system can be divided into the power consumed by a fan to drive the air through the filters (P_f_) and the input power for sterilization (P_s_). The electric energy consumption^[21]^ can be calculated as follows:

$P_{f}=\frac{\Delta P\times Q}{\eta}$

$(12)$

$P_{S}=U\times I$

$(13)$

$P_{t}=P_{f}+P_{s}$

$(14)$

where ΔP is the pressure drop across the filter in Pascals (Pa), 𝑄 is the airflow rate through the filter in cubic meters per second, 𝜂 is the efficiency of the fan (71%).

1. The costs of HEPA+UVGI, ESP, and LIG are available at Amazon and XFnano.

**Note S1** Electrical property of BCNMs.

The heat generation (*Q_J_*) of BCNMs obeys Joule’s law,^[23]^

$\frac{dQ_{J}}{dt}=\frac{dW}{dt}=P=\frac{U^{2}}{R}$

$(1)$

where *U* is the applied voltage, *t* is the heating time, *R* is the resistance of BCNMs under this voltage, *W* is the electric work, and *P* is the electric power. A steady temperature can be reached when the heat of joule heating and convective heat transfer (*Q_c_*) reaches a dynamic balance,

$\frac{dQ_{c}}{dt}=hA\left( T_{s}-T_{e} \right)$

$(2)$

where h and A are the heat transfer coefficient and surface area of BCNMs. Therefore, the steady temperature (*T_s_*) of BCNMs is:

$T_{s}=\frac{1}{h}\frac{U^{2}}{RA}+T_{e}=\frac{1}{h}\frac{P}{A}+T_{e}=\frac{1}{h}q+T_{e}$

$\left( 3 \right)$

where *q* is the power density; 1/*h* represents the electrothermal conversion efficency.

**Note S2** Heat transfer mechanism of BCNMs.

BCNM can generate more concentrated heat due to its two advantages. First, BCNM can generate more efficient Joule heat. Second, BCNM has less heat loss. Specifically,

BCNM is an electrothermal material with nanoscale pores. The addition of highly conductive PPy makes the Joule heat generation of the membrane material more uniform and efficient at the three-dimensional structural level. In addition, BCNM has low thermal conductivity of only 0.08 W m^-1^ K^-1^. From a theoretical analysis, the thermodynamic behavior of electrothermal materials follows the law of energy conservation at steady state (temperature stabilizes):

where $P_{Joule}$ is Joule heating power. $P_{conduction}$, $P_{convection}$ and $P_{radiation}$ are thermal energy lost per unit time via conduction, convection, and radiation. Conduction loss $P_{conduction}$ is directly tied to thermal conductivity $\kappa$:

where $A$ is the cross-sectional area for heat transfer. $\Delta T$ is the temperature difference between heater and heat sink. $L$ is the heat transfer path length. Hence, if $\kappa$ decreases, $P_{conduction}$ significantly reduces. To balance Eq. 4, $P_{convection}$ and $P_{radiation}$ must increase. $P_{convection}\approx hA(T-T_{0})$ and $P_{radiation}=\epsilon\sigma A(T^{4}-T_{0}^{4})$ nonlinearly increase with solid surface temperature $T$, therefore, $T$ will increase as $\kappa$ decreases.

We also used numerical simulation to compare the temperature rise of high thermal conductivity materials (Carbon nanotubes, $\kappa$ is 2000W m^-1^ K^-1^ ) and BCNM ($\kappa$ is 0.08 W m^-1^ K^-1^) at the transient state. The simulation methods and boundary conditions can be found in section II of Note S3, Supporting information. It can be seen from the Figure S17 that at t=200 s, the maximum temperature of the BCNM membrane can reach 149 ℃, which is 34.2% higher than the temperature of the high thermal conductivity material. Therefore, these directly prove that BCNM can generate more concentrated heat to achieve efficient energy utilization.

**Note S3** Simulation methodology

I. The filtration performance simulation of homogeneous fibrous membrane and hierarchical fibrous membrane

The filtration effect of nanofibrous rmembrane on air-containing particles is simulated using COMSOL Multiphysics software based on experimental parameters (Table S4). We compared the filtration performance of the homogeneous and hierarchical fibrous models. In the numerical simulation, we assume that the filter membrane is composed of three groups of fibers, and each group of fibers consists of two layers of fibers of the same type that are perpendicular to each other. In addition, each fiber is cylindrical. In homogeneous mode, the filter membrane is composed of three groups of PAN nanofiber, and their diameter, fiber spacing (spacing between fibers in the same layer) and layer spacing (the distance between two adjacent layers of fibers) are shown in Table S4. In hierarchical mode, the filter membrane is arranged from top to bottom in the order of PPy fiber group, PAN fiber group and PET fiber group. The computational domain is a cylinder with a height of 100 μm and a diameter of 80 μm. The filter membrane is located in the center of the cylinder.

The single-phase ﬂow (SPF) laminar module is employed to calculate the steady-state flow field, which is based on the Navier-Stokes equations.

$\rho\left( \boldsymbol{u}\cdot\nabla\right)\boldsymbol{u}=\nabla\cdot\left[ p\boldsymbol{I}+\boldsymbol{K} \right]+\boldsymbol{F}$

$(6)$

$\nabla\cdot\left( \rho\boldsymbol{u} \right)=0$

$(7)$

where *ρ* is the density, ***u*** is the velocity vector, *p* is =pressure, ***I*** is the identity matrix, ***K*** is the viscous stress tensor, and ***F*** is the volume force vector. In the SPF module, the fluid is assumed to be air, which is a compressible fluid (Ma<0.3). The boundary condition of the inlet (the top surface of the cylinder) is velocity (normal inflow velocity: 0.138 m s^-1^) and that of the outlet (the bottom surface of the cylinder) is pressure.

After that, the ﬂuid-ﬂow particle tracking (FPT) module is employed to calculate the transient motion of particles in the flow field. Here, the particle motion is driven by the drag force from the fluid. The boundary condition of the inlet is to release 10,000 particles per time. The density of particles is 2200 kg m^-3^, and the diameter of particles is 300 nm. Besides, the solid surfaces are set as freezing walls, which means the particle's position no longer changes after it hits the wall, and the particle’s velocity remains the same value as when the particles hit the wall. The grid number of the homogeneous and hierarchical modes is 1721364 and 2366518 respectively.

**Table S4** Material parameters in the filtration performance simulation of homogeneous fibrous membrane and hierarchical fibrous membrane

| Fiber group | Parameters | Homogeneous mode | Hierarchical mode |
| --- | --- | --- | --- |
| The top fiber group | Material | PAN | PPy |
|  | Fiber diameter (μm) | 0.47 | 0.244 |
|  | Fiber spacing (μm) | 0.8 | 0.44 |
|  | Layer spacing (μm) | 1 | 1 |
| The middle fiber group | Material | PAN | PAN |
|  | Fiber diameter (μm) | 0.47 | 0.47 |
|  | Fiber spacing (μm) | 0.8 | 0.8 |
|  | Layer spacing (μm) | 1 | 1 |
| The bottom fiber group | Material | PAN | PET |
|  | Fiber diameter (μm) | 0.47 | 12.7 |
|  | Fiber spacing (μm) | 0.8 | 9.6 |
|  | Layer spacing (μm) | 1 | 13 |

II. Simulation of temperature distribution and heat transfer situation of self-heating BCNM (S-Model) and externally heated HEPA filters (E-Model) in tubes

In the numerical simulation comparing the self-heating membrane and the externally heated membrane, we considered the heating of the air flowing through the filter membrane by the heating source, its own heat dissipation, and radiation heat transfer. A 2D axisymmetric model (Figure S24) is used to further simplify the model and speed up the calculation. The size of the geometric model in the numerical simulation is completely consistent with the experimental setup. Air flows in from the top inlet, passes through the filter membrane (BCNM or HEPA), and flows out from the outlet. In the S-model, the filter membrane is BCNM, which is self-heating. In the E-model, the filter membrane is HEPA, which is heated by a heater surrounding the outside of the pipe. Here, the filter membrane is simplified to a porous medium region with a porosity of 0.84. The thermal field takes into account heat conduction, heat convection and heat radiation.


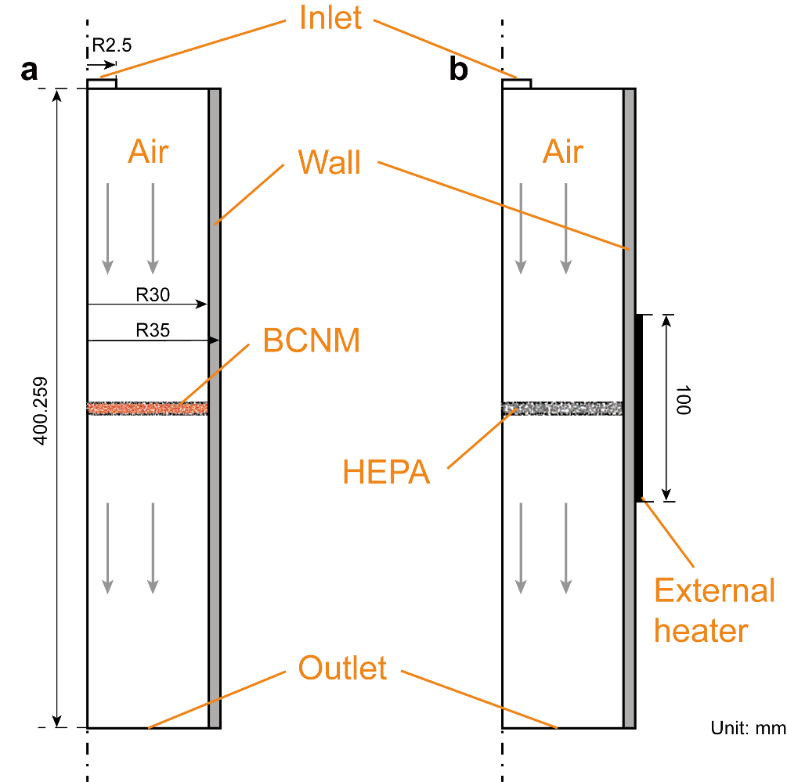


**Figure S24.** Computational geometry model for simulation of temperature distribution and heat transfer situation of self-heating BCNM (S-Model) and externally heated HEPA filters (E-Model) in tubes.

The boundary conditions are as follows. 1) Flow Field: The inlet is defined as the velocity inlet and the pressure of outlet is 0 Pa. The boundary condition of the wall is no slip wall. The flow in porous media is Darcian flow. 2) Thermal Field: The inlet air temperature is 303 K. The BCNM and external heater are added to the simulation as heat sources, which come from electric heating (Q=UI, U is voltage, and I is current). The whole cylindrical tube wall is cooled by external natural convection. The surfaces of BCNM, HEPA and external heater are non-transparent surface. Other surfaces are transparent. The specific parameters are listed in Table S5.

**Table S5** Material parameters in simulation of self-heating BCNM (S-Model) and externally heated HEPA filters (E-Model) in tubes

| Material properties | Value |
| --- | --- |
| Thermal conductivity (W m^-1^ K^-1^) | 0.08 |
| Specific heat capacity (J kg^-1^ K^-1^) | 1000 |
| Density (kg m^-3^) | 1184 |
| Thickness (mm) | 0.259 |
| Porosity | 0.84 |
| Heat source (W cm^-2^) | 0.28 (For BCNM)  0.37 (For HEPA) |

The flow in the fluid domain is laminar due to Re < 2000. The flow field is calculated as Eq. 8.

$\rho\frac{\partial\boldsymbol{u}}{\partial t}+\rho\left( \boldsymbol{u}\cdot\nabla\right)\boldsymbol{u}=\nabla\cdot\left[ p\boldsymbol{I}+\boldsymbol{K} \right]+\boldsymbol{F}$ (8)

In the heat transfer (HT) module, the temperature is solved as the Eq. 9 and 10. The convection includes heat exchange between the internal fluid and the tube wall, and natural convection between the tube wall and the outside air, both of which are solved according to$hA(T-T_{0})$. Besides, radiative heat transfer occurs between a non-transparent solid surface and the environment, satisfying$\varepsilon\sigma A(T^{4}-T_{0}^{4})$, where $\varepsilon$=0.8.

$\rho C_{P}\frac{\partial T}{\partial t}+\rho C_{P}\boldsymbol{u}\cdot\nabla T+\nabla\cdot\boldsymbol{q}=Q$

$\left( 9 \right)$

$\boldsymbol{q}=-k\nabla T$

$(10)$

where *C_p_* is the specific heat capacity at constant pressure, *Q* is the heat source, and ***q*** is the heat flux by conduction.

In the meshing part, we chose the physics-controlled mesh, which includes the boundary layer of the fluid domain and the mesh refinement of the narrow area of the film. After completing the grid independence verification (Figure S25), we selected 23692 grids for calculation. Finally, the transient solution is used in the study, and the temperature distribution at the time *t*=200 s is selected as the result.

**
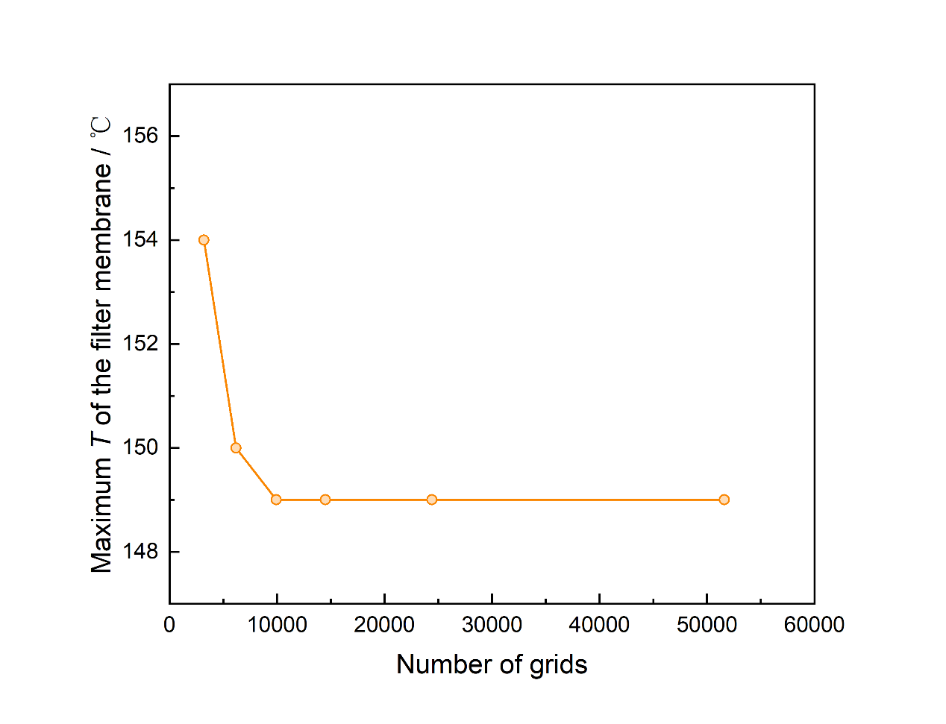
**

**Figure S25.** Grid independence study.

III. The room filtration performance simulation

A 5×5×5 m^3^ room (Figure S23) was used to simulate the filtration performance of BCNM-purifier. The BCNM-purifier with a diameter of 20 cm is placed in the center of the room. When it is turned on, it can absorb two particles that simulate human exhalation. In addition, natural ventilation inlets and outlets are arranged above and below the room to ensure normal circulation of indoor air. The governing equation of the fluid domain is shown in Eq. 8.

The boundary conditions are as follows:

1. The room has an indoor natural ventilation system. The velocity of the inlet is 1 m s^-1^ and the outlet is set as 0 Pa.
2. Each dummy releases 5,000 particles each time and the release time is 1 s. The particles are mainly composed of water and have a diameter of 10 μm.
3. The BCNM-purifier is installed on the table in the middle of the room, and the air exhaust pressure difference is -76 Pa. The air filter is open in the testing group and that in the blank group is closed.
4. Except for the outlets, which are *disappear* boundaries, all other walls are *freeze* boundaries.

**Reference**

[1] Z. Niu, C. Xiao, J. Mo, L. Zhang, C. Chen, Investigating the Influence of Metal–Organic Framework Loading on the Filtration Performance of Electrospun Nanofiber Air Filters, *ACS Appl. Mater. Interfaces* **2022**, *14*, 27096.

[2] S. Hartati, A. Zulfi, P. Y. D. Maulida, A. Yudhowijoyo, M. Dioktyanto, K. E. Saputro, A. Noviyanto, N. T. Rochman, Synthesis of Electrospun PAN/TiO2/Ag Nanofibers Membrane As Potential Air Filtration Media with Photocatalytic Activity, *ACS Omega* **2022**, *7*, 10516.

[3] H. Dai, X. Liu, C. Zhang, K. Ma, Y. Zhang, Electrospinning Polyacrylonitrile/Graphene Oxide/Polyimide nanofibrous membranes for High-efficiency PM2.5 filtration, *Sep. Purif. Technol.* **2021**, *276*, 119243.

[4] S. Guo, B. Yu, A. Ahmed, H. Cong, Y. Shen, Synthesis of polyacrylonitrile/polytetrahydropyrimidine (PAN/PTHP) nanofibers with enhanced antibacterial and anti-viral activities for personal protective equipment, *J. Hazard. Mater.* **2022**, *424*, 127602.

[5] Q. F. Lim, R. C. C. Yap, C. P. Teng, J. C. C. Yeo, M. Y. Tan, J. P. W. Toh, Q. Zhu, W. Thitsartarn, C. He, S. Liu, J. Kong, Electrospray-on-Electrospun Breathable, Biodegradable, and Robust Nanofibrous Membranes with Photocatalytic Bactericidal Activity, *ACS Appl. Nano Mater.* **2023**, *6*, 1828.

[6] J. Lee, J. Bae, D.-Y. Youn, J. Ahn, W.-T. Hwang, H. Bae, P. K. Bae, I.-D. Kim, Violacein-embedded nanofiber filters with antiviral and antibacterial activities, *Chem. Eng. J.* **2022**, *444*, 136460.

[7] H. Lee, S. Jeon, Polyacrylonitrile Nanofiber Membranes Modified with Ni-Based Conductive Metal Organic Frameworks for Air Filtration and Respiration Monitoring, *ACS Appl. Nano Mater.* **2020**, *3*, 8192.

[8] V. Kadam, Y. B. Truong, C. Easton, S. Mukherjee, L. Wang, R. Padhye, I. L. Kyratzis, Electrospun Polyacrylonitrile/β-Cyclodextrin Composite Membranes for Simultaneous Air Filtration and Adsorption of Volatile Organic Compounds, *ACS Appl. Nano Mater.* **2018**, *1*, 4268.

[9] C. Liu, P.-C. Hsu, H.-W. Lee, M. Ye, G. Zheng, N. Liu, W. Li, Y. Cui, Transparent air filter for high-efficiency PM2.5 capture, *Nat. Commun.* **2015**, *6*, 6205.

[10] S. Zhang, H. Liu, X. Yin, J. Yu, B. Ding, Anti-deformed Polyacrylonitrile/Polysulfone Composite Membrane with Binary Structures for Effective Air Filtration, *ACS Appl. Mater. Interfaces* **2016**, *8*, 8086.

[11] S. Zhang, N. Tang, L. Cao, X. Yin, J. Yu, B. Ding, Highly Integrated Polysulfone/Polyacrylonitrile/Polyamide-6 Air Filter for Multilevel Physical Sieving Airborne Particles, *ACS Appl. Mater. Interfaces* **2016**, *8*, 29062.

[12] H. Zhang, S. Fang, Z. Wu, S. R. Islam, X. Qin, PAN/PAN-FPU Double-Layer Composite Nanofiber Air Filter Fabricated by Electrospinning for Filtering Oil and Salt Particles, *ACS Appl. Nano Mater.* **2023**, *6*, 7619.

[13] Y.-I. Kim, M.-W. Kim, S. An, A. L. Yarin, S. S. Yoon, Reusable Filters Augmented with Heating Microfibers for Antibacterial and Antiviral Sterilization, *ACS Appl. Mater. Interfaces* **2021**, *13*, 857.

[14] L. Issman, B. Graves, J. Terrones, M. Hosmillo, R. Qiao, M. Glerum, S. Yeshurun, M. Pick, I. Goodfellow, J. Elliott, A. Boies, Filtration of viral aerosols via a hybrid carbon nanotube active filter, *Carbon* **2021**, *183*, 232.

[15] A. Reimers, A. Bouhanguel, E. Greve, M. Möller, L. M. Saure, S. Kaps, L. Wegner, A. S. Nia, X. Feng, F. Schütt, Y. Andres, R. Adelung, Multifunctional, self-cleaning air filters based on graphene-enhanced ceramic networks, *Device* **2023**, *1*, 100098.

[16] M. G. Stanford, J. T. Li, Y. Chen, E. A. McHugh, A. Liopo, H. Xiao, J. M. Tour, Self-Sterilizing Laser-Induced Graphene Bacterial Air Filter, *ACS Nano* **2019**, *13*, 11912.

[17] S. Han, J. Kim, Y. Lee, J. Bang, C. G. Kim, J. Choi, J. Min, I. Ha, Y. Yoon, C.-H. Yun, M. Cruz, B. J. Wiley, S. H. Ko, Transparent Air Filters with Active Thermal Sterilization, *Nano Lett.* **2022**, *22*, 524.

[18] J. Xiong, A. Li, Y. Liu, L. Wang, X. Qin, J. Yu, Multi-Scale Nanoarchitectured Fibrous Networks for High-Performance, Self-Sterilization, and Recyclable Face Masks, *Small* **2022**, *18*, 2105570.

[19] S. Hwang, J. Roh, W. M. Park, Comparison of the relative performance efficiencies of melt-blown and glass fiber filter media for managing fine particles, *Aerosol Sci. Technol.* **2018**, *52*, 451.

[20] F. Vranay, L. Pirsel, R. Kacik, Z. Vranayova, Adaptation of HVAC Systems to Reduce the Spread of COVID-19 in Buildings, *Sustainability* **2020**, *12*, 9992.

[21] P. Wang, J. Liu, C. Wang, Z. Zhang, J. Li, A holistic performance assessment of duct-type electrostatic precipitators, *J. Clean. Prod.* **2022**, *357*, 131997.

[22] A. C. K. Lai, A. C. T. Cheung, M. M. L. Wong, W. S. Li, Evaluation of cold plasma inactivation efficacy against different airborne bacteria in ventilation duct flow, *Build. Environ.* **2016**, *98*, 39.

[23] Y. Zhang, H. Ren, H. Chen, Q. Chen, L. Jin, W. Peng, S. Xin, Y. Bai, Cotton Fabrics Decorated with Conductive Graphene Nanosheet Inks for Flexible Wearable Heaters and Strain Sensors, *ACS Appl. Nano Mater.* **2021**, *4*, 9709.
